# Shower effect of a rainfall onset on the heat accumulated during a preceding dry spell

**DOI:** 10.1038/s41598-019-43437-7

**Published:** 2019-05-07

**Authors:** Rajarshi Das Bhowmik, Bihu Suchetana, Mengqian Lu

**Affiliations:** 1 Department of Civil Engineering, Indian Institute of Science, Bangalore, 560012 India; 2 Interdisciplinary Center for Water Research, Indian Institute of Science, Bangalore, 560012 India; 3 Department of Civil and Environmental Engineering, The Hong Kong University of Science and Technology, Clear Water Bay, SAR Hong Kong

**Keywords:** Climate sciences, Hydrology

## Abstract

Popular perception claims that rain following a hot day brings relief, indicating a bio-meteorological perspective of ‘rainy’ forecasts. However, the hypothesis has rarely been examined on India which experiences distinct pre- and post-monsoon seasons with continuous dry days, occasionally interrupted by thunderstorms or cyclones. The current study analyzes 54 years of observed daily meteorological records across India to assess the impact of shower effect, defined as the amount of change in the temperature on the first day of a wet spell that succeeds a dry spell. Nine combinations of low to high probability rainfall events on the first day of a wet spell and short to prolonged dry spell categories are evaluated. Results indicate that the north, the northeastern, and the eastern states of India witness a decrease in the maximum and minimum temperatures, up to 5 °C during the pre-monsoon season while mostly exhibiting a statistically insignificant long-term temporal trend. During the post-monsoon season, a rainfall event decreases the maximum temperature, providing significant relief by reducing the heat index (HI) warning from ‘Caution’ to ‘Normal’, but is unable to lower the HI warning from ‘danger’ during the pre-monsoon season.

## Introduction

It is generally believed that rainfall leads to a decrease in the surface and the near-surface temperature. For tropical countries like India, rainfall not only dictates water resource operations, agricultural yield, food security, and disease outbreak, but more immediately brings a general sense of relief from the heat^1–3^. In short, rainfall seems to have a profound bio-meteorological and psychological impact, even if the rainfall event doesn’t lead to lower temperatures. Whether or not the perception regarding rain’s impact on the decreasing temperature is true, a weather forecast of rainfall on the next day following a dry spell (days with no rainfall) is welcome news^4^. This role of rain resembles a cold shower a person takes after a long, dry and hot day; therefore, the current study proposes to call the weather phenomenon the shower effect. However, to the best of our knowledge, the major scientific questions related to the shower effect were never investigated- does rain really bring down the temperature? If yes, by what magnitude? Along with these two major research questions, the current study also investigates the impact of rain on bio-meteorological relief and discusses the potential inclusion of the shower effect on weather forecasting.

**Figure 1:**
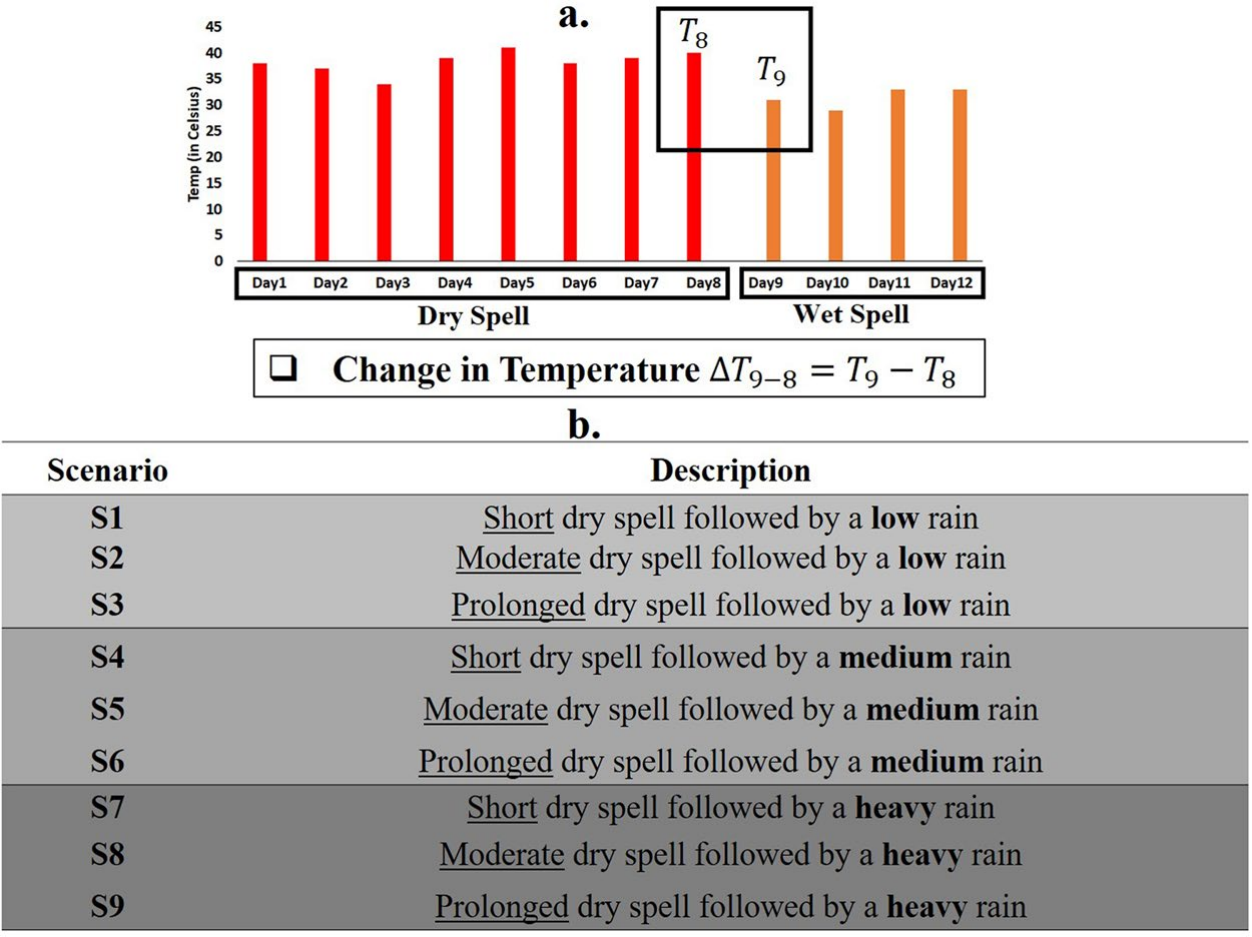
(**a**) Schematic demonstrates the approach to calculate the decrease in the temperature on the first day of a wet spell that succeeds a dry spell, and (**b**) List of nine scenarios combining three categories of dry spell lengths with three types of rain on the first day of a wet spell. The study calculates the change in the temperature under each scenario.

Earlier studies related to wet days (days with more than 2.5 mm rainfall) and dry day sequences investigated the amount of rainfall on a wet day, which bears significance in water resources and climate change impact assessment^5^. Similar studies performed long-term trend analysis, examined the impact of climate change, simulated rainfalls under different climate change scenarios, characterized drought conditions etc.^6,7^. However, the rise in temperature on either the first or on a later day of a dry spell succeeding a wet spell, and the decline in the temperature on the first wet day after a dry spell were neither investigated nor modelled. The expected decline in temperature on a day within a wet spell is not significant since continuous rainy days (often resulted from deep depressions) are expected to keep the temperature within comfortable bounds. Similarly, we note that the rise in the temperature on the first or a later day of a dry spell is not directly related to rainfall, since the absence of rain, seasonality, wind speed and wind direction, albedo, air-quality conditions and other local-scale drivers typically force a rise in daily temperature^8–11^. Considering these factors, we accept the change in the temperature on the first day of a wet spell succeeding a dry spell as our variable of interest. Further, we assume that the change in the temperature is directly associated with the rainfall amount on the first day of a wet spell. Figure 1(a) demonstrates the approach to calculate the change in the temperature. Similar to previous research on stochastic weather generators, we assume that wet and dry spells follow a Markovian process, where wet and dry spells always follow each other^12^. The current study categorizes rainfall events as low, medium and heavy rains, based on the marginal probability of an amount (in mm/day) of daily rainfall. Similarly, dry spells are divided into three groups-short, moderate and prolonged, based on the length of a dry spell^13–15^. Shower effect is investigated under nine scenarios (S1 to S9): an exhaustive combination of three types of rainfall events and three categories of dry spells. A description of these nine scenarios is presented in Fig. 1b. Change in the temperature on the first day of a wet spell that succeeds a dry spell is considered as the variable of interest (hereafter referred to as change in the temperature), whose value is estimated for nine scenarios. Details regarding dry spell lengths and rainfall events are provided in the Methodology section. Note that, three dry day categories remain constant across all grid cells. However, rainfall percentiles are estimated for individual grid cells; therefore, rainfall thresholds differ from cell to cell, to reflect spatial variability (see Fig. S1 in Supplementary Information for threshold values). In addition, rainfall threshold values at a given grid cell vary seasonally since the thresholds are calculated based on seasonal rainfall records. Considering these reasons, although the basic definitions of the nine scenarios remain the same across grid cells, the underlying rainfall thresholds account for various climate conditions under the same scenario based on the location of a grid cell and the season of the year. We do not estimate the joint probability of each of the nine scenarios or the marginal probabilities of the dry spell events since they are expected to vary substantially over space and time. One former study^13^ investigated the spatio-temporal patterns of rainfall events across India based on a similar classification technique, and reported that barring two southern states (which receive heavy rainfall during October and December), the rest of the country receive heavy rainfall resulting from the southwest monsoon.

Shower effect is expected to be pronounced for India, as the country experiences a prominent monsoon season, during which it receives a majority of its rainfall, and beyond which rain events are infrequent^16–18^. Additionally, the pre- and the post-monsoon seasons (March-April-May and October-November-December respectively) witness the persistence of extreme high temperature or pre-monsoon heat waves^19^, occasionally interrupted by rains from scattered thunderstorms, cyclone or deep depressions^20,21^. It is important to note that it is difficult to examine the shower effect if the annual rainfall is uniformly distributed throughout the year (for example: East coast of the United States). This is because some of the nine scenarios listed above may not have the sampling variability to carry out a detailed analysis. In other words, the clear presence of a pre- and post- monsoon heat in India, interrupted by monsoon showers, makes it the ideal location to study the impact of the shower effect.

**Figure 2:**
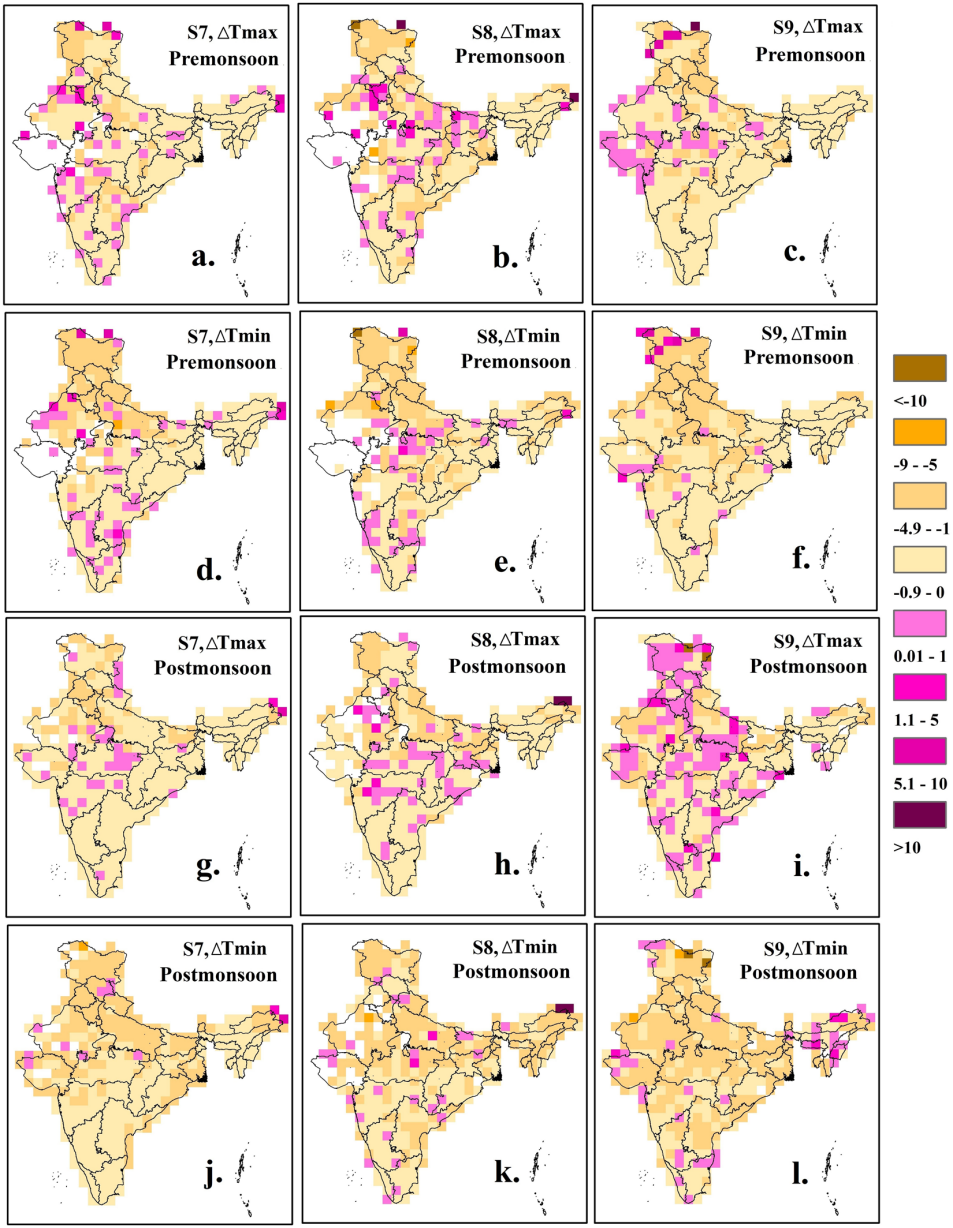
Average change in the temperature in °C (*Tmax* and *Tmin*) across India under three different scenarios (S7, S8 and S9) for the pre- and post-monsoon seasons, 1951–2004.

**Figure 3:**
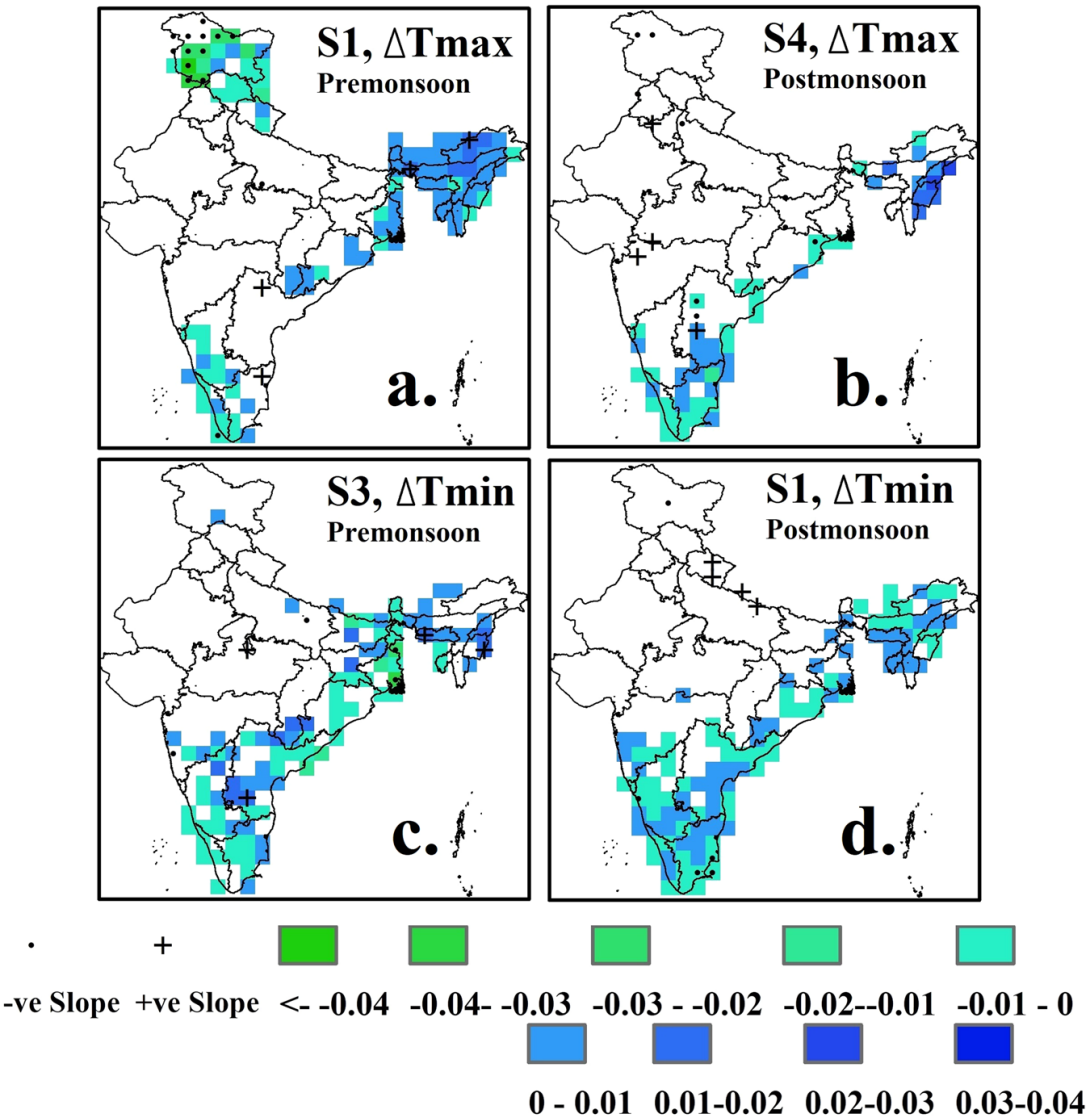
Temporal trend in the change in temperature (*Tmax* and *Tmin*) for the Pre- and post-monsoon seasons, 1951–2004. Scenarios S1 and S4 (S3 and S1) are shown for the change in *Tmax* (drop in *Tmin*). Grid points that exhibit either statistically significant positive or statistically significant negative trends, based on the Mann-Kendall test, are marked. Temporal trend is estimated following Sen slope estimator.

We obtained gridded (1° × 1°) observational precipitation (in mm/day), maximum temperature (in °C) and minimum temperature (in °C) data on a daily scale for the period 1/1/1951-31/12/2004 from the Indian Meteorological Department (IMD)^21^. On each grid cell, change in the maximum temperature (*Tmax*) and in the minimum temperature (*Tmin*) during the pre- and post-monsoon seasons are estimated under nine hydro-meteorological scenarios. Long-term average of the estimated changes in *Tmax* and *Tmin* on each grid cell is presented in Fig. 2 for three selected scenarios- S7, S8 and S9 (detailed results are provided in Supplementary Figs S2 to S7). Decreases in *Tmax* and *Tmin* throughout the country becomes more prominent as the length of the dry spell increases. In general, decrease in *Tmin* is slightly higher in magnitude than the decrease in *Tmax*. This phenomenon can be linked to two weather conditions, detailed as follows. First, a prolonged dry spell (particularly during the pre-monsoon season) increases the temperature substantially, leading to persistent high temperatures (see Supplementary Fig. S8). This anomalous temperature is easily restored to its climatological state even by a small amount of rainfall. On the other hand, a short duration dry spell is not capable of increasing the temperature above its climatological mean. Therefore, rainfall following a short duration dry spell does not reduce temperature as much as compared to a rainfall event following a prolonged dry spell. Second, on multiple occasions, prolonged dry-spells have ended with deep-depression and cyclones. These large-scale atmospheric conditions have the potential to decrease the temperature substantially. The second possible condition requires continuous monitoring of large scale atmospheric conditions and local weather conditions which are beyond the scope of the current study. During the pre-monsoon season, heavy rain on the first day of a wet spell, irrespective of the length of the dry spell it succeeds, results in a larger decrease in the temperature as compared to a low rain on the first day of a wet spell. Magnitudes of the decrease in the temperature are substantial (up to 5 °C) for the northern and the eastern states like Uttar Pradesh, Bihar, West Bengal and Punjab where scattered thunderstorms are common during the pre-monsoon season. During the post-monsoon season, a rainfall event has least impact on the decrease in *Tmax*; therefore, given a dry spell category (short/moderate/prolonged) *Tmax* values on the last day of a dry spell and on the first day of a wet spell remains almost same. In fact, for some grid cells, *Tmax* might even increase following a rainfall event on the first day of a wet spell. However, the decrease in *Tmin* on the first day of a wet spell is substantial during the post-monsoon season, and its magnitude increases as either the length of the dry spell increases or the amount of rainfall on the first day of a wet spell increases (Fig. 2(j), Fig. 2(k), and Fig. 2(l)). Post-monsoon rainfall events in India often result from cyclones and deep depressions^19^ which affect a larger part of the country than scattered thunderstorms, which typically impact only a few grid cells (for example: grid cells covering Uttar Pradesh, a state in the northern part of India) at a time. Therefore, a significant decrease in the *Tmin* (more than 5 °C) for the post-monsoon season can be seen over the western, the northern and the eastern part of the country. Note that, irrespective of rain or no rain, diurnal temperature variation is common during the post-monsoon season^22^; therefore, a decrease in the *Tmin* on the first day of a wet spell can be partly attributed to the diurnal temperature variation. We note that seasonality in diurnal temperature does not have an impact on the calculation of nine scenarios. Despite large magnitudes of decrease in the temperature across multiple hydro-climate regions across the country, the variable exhibits either small or no long-term temporal trend as indicated by the results of Man-Kendall test with annually averaged values, and the Sen slope values (Fig. 3; detailed results are provided in Supplementary Figs S9 to S12). During the pre- and the post-monsoon seasons, a small but statistically significant negative (positive) trend in the change in *Tmax* exists across the southern (the northeast) India, indicating the magnitude of the variable is increasing (decreasing) over the years. However, the long-term trends across most of the grid cells are not statistically significant. The interpretation of the results is based on the assumption that annually averaged values of the change in the temperature are always negative. During the pre-monsoon season, statistically significant negative trends in the change in *Tmax* is seen for the northern state of Jammu & Kashmir under scenario S1. Results related to *Tmin* are similar to that of *Tmax*, with statistically insignificant negative trends existing across the coastal southeast and the northeast. Note that there are only few grid cells across India where the temporal trends are statistically significant. Therefore, it is necessary to investigate whether temporal trends on these grid cells lead to a global significance or not. To examine for the global significance of the locally rejected null hypothesis (i.e., non-existence of temporal trend based on p-value lesser than or equal to 0.025 for a two-sided test), the current study follows a field significance test based on false discovery rate^23,24^. From the results of the field significance test it is clear that none of the scenarios during the pre- or the post-monsoon seasons exhibits a regional significance of locally significant temporal trends in the change in the temperature (see Supplementary Information, Tables S1 and S2).

**Figure 4:**
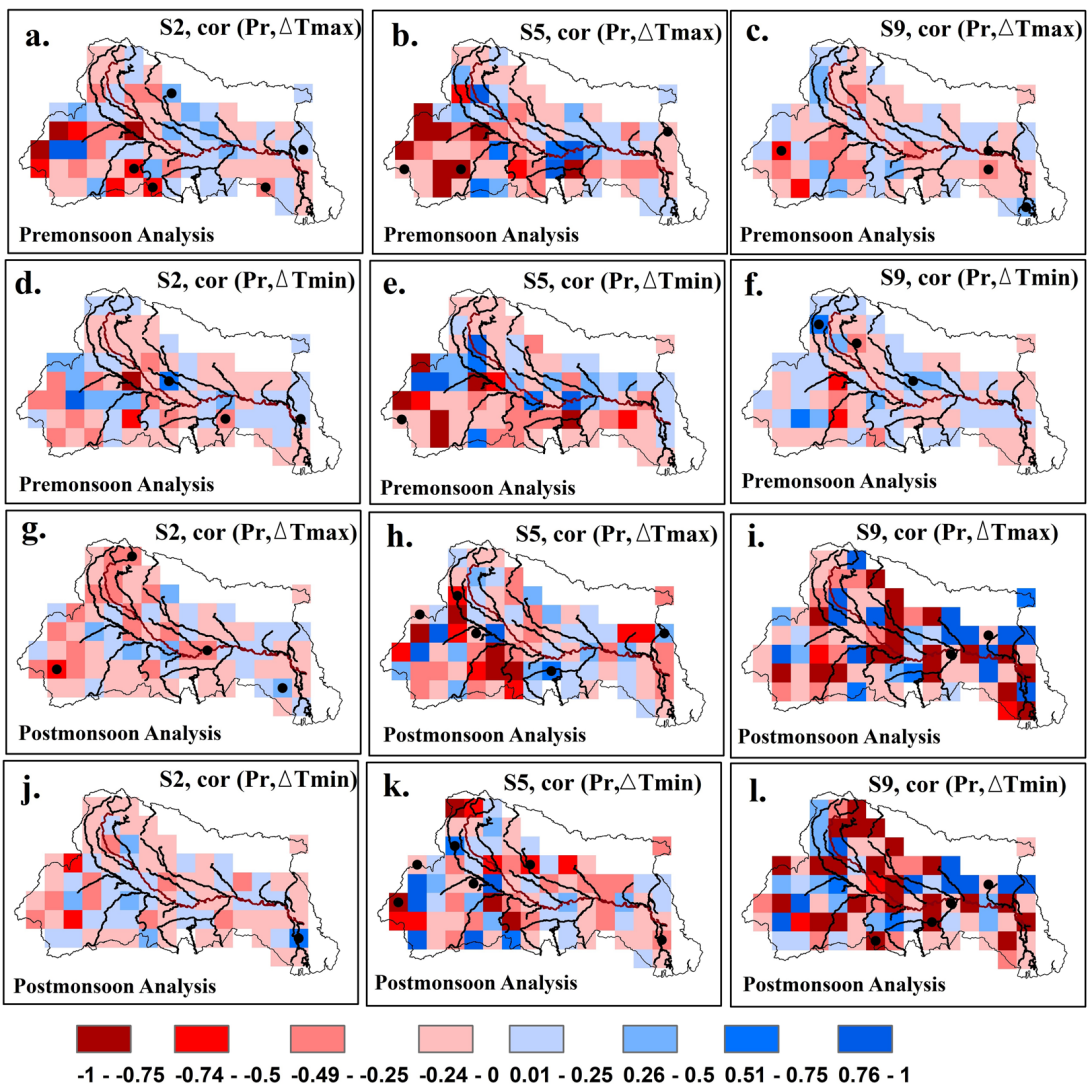
Maps show Kendall’s rank correlation coefficient between the change in the temperature (*Tmax* and *Tmin*) and the rainfall amount on the wet day succeeding a dry spell under scenarios S2, S5 and S9 for the pre- and the post-monsoon seasons. Results are presented over the Ganga Basin. Statistically significant grid cells where p-values are lesser than 0.05 are marked with black circles.

**Figure 5:**
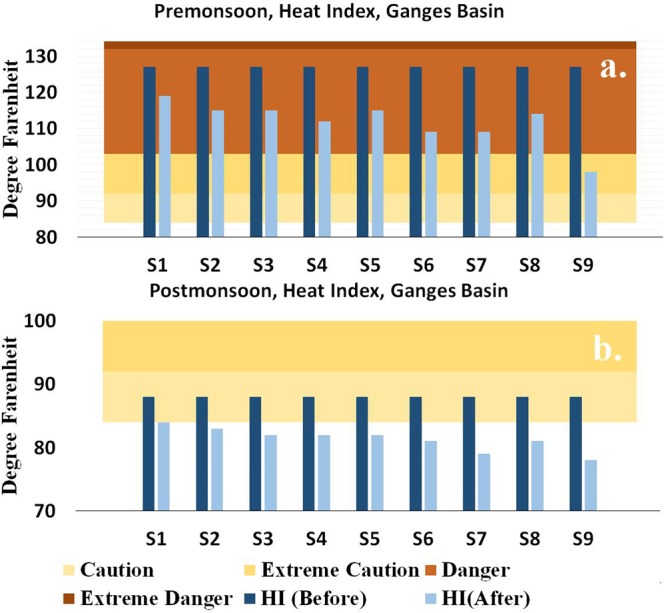
Heat Index (HI) values in °F on the last day of the dry spell HI (Before) and on the first day of a wet spell that succeeds the dry spell HI (After) are presented as the bars. Background colors represents four HI warnings, ranging from caution to Extreme danger. For the current analysis, Heat Index is calculated using the spatially average decrease in the *Tmax* values, and spatially averaged relative humidity values over the Ganga basin. Gridded relative humidity, precipitation and maximum temperature values are obtained from the NCEP/NCAR reanalysis project (CDAS-I).

To further analyze the correlation between the amount of rainfall and the change in the temperature on the first day of a wet spell (Fig. 4) and the heat index estimation (Fig. 5; detail results are provided in Supplementary Figs S13 to S18), we focus on the Ganga Basin: the northern and the eastern India that experience the largest magnitudes of the shower effect and witness frequent thunderstorms, deep depressions and cyclones. The current study estimates Kendall’s rank correlation coefficient between the change in the temperature and the amount of rainfall on the wet day that succeeds a dry spell. Grid cells where the rank correlation coefficients are statistically significant (i.e., p-value less than or equal to 0.05) are marked with a black circle. In addition, field significance tests based on false discovery rate^23,24^ are carried out to examine the global significance of locally rejected null hypothesis (local null hypothesis states that change in the temperature and the amount of rainfall at a grid cell are uncorrelated). In general, a strong but negative correlation between rainfall amount and the change in the temperature is found across the Ganga basin when a moderate dry spell ends with a rain. During the pre-monsoon season, the correlation is weakest (strongest) when a rainfall event follows a prolonged (moderate) dry spell. It indicates that rainfall amounts do not significantly impact the change in temperature when dry spell duration is longer than 14 days. This condition can be partially explained from our earlier findings that mean temperature during a dry spell increases with the increase in dry spell duration (Fig. S8), and the decrease in temperature become more prominent as the dry spell duration increase (Fig. 2). Therefore, the correlation for short dry spell duration is weaker than the correlation for moderate dry spell duration. However, the finding related to the difference in correlations between moderate and prolonged dry spells remains unexplained. During both seasons, the correlation conditioned on the dry spell duration (either short or moderate or prolonged) increases as the amount of rain increases from low to heavy. However, during the post-monsoon season, the correlation between change in the temperature and a rainfall event that follows either a prolonged or a moderate dry spell are similar. The concordance between rain and temperature during the pre-monsoon is slightly stronger for *Tmax* than *Tmin*. We note that only a few grid cells under any of the nine scenarios exhibit statistically significant Kendall’s rank correlation coefficients. Typically, only 10% of the grid cells across the Ganga basin exhibit statistically significant negative correlation. A field significance tests based on false discovery rate (see Supplementary Information Tables S3 and S4) confirm that there is no regional significance of locally significant correlations except for the scenario S4 of pre-monsoon *Tmin* (scenario S4 is defined as medium rain succeeding a moderate dry spell). Hence, spatial correlation patterns between change in the temperature and rainfall amount are not significant.

In a final analysis, we estimate the impact of the decrease in *Tmax* on the bio-meteorological relief by calculating the heat index (HI) for the Ganga Basin. The current analysis considers gridded relative humidity at 1000 mb from the NOAA/NCAR reanalysis products (CDAS-1), since gridded observed relative humidity values are not available from IMD. To maintain consistency in data source, the current analysis also obtains gridded daily precipitation and maximum temperature datasets from the NOAA/NCAR reanalysis products (CDAS-1). Following that, the current study applies a similar methodology under nine scenarios to estimate the change in *Tmax*. We calculate the heat index on the last day of a dry spell (labelled as ‘HI (Before)’ in Fig. 5) and on the first day of a wet spell (labelled as ‘HI (After)’ in Fig. 5) using relative humidity, *Tmax* and the change in *Tmax*. During the pre-monsoon season, a heavy rain following a short/moderate/prolonged dry spell is most effective in reducing the heat index warning as compared to a low or a medium rain (Fig. 5). However, during the post-monsoon season, any amount of rain, in general, is sufficient to provide relief by reducing the warning from ‘Caution’ to ‘Normal’. We note that with the exception of S9 (heavy rainfall after a prolonged dry spell), none of the other rainfall scenarios during the pre-monsoon season can reduce the warning below a ‘danger’ category. The current study also calculates the heat index for the Ganga Basin using daily precipitation and daily maximum temperature data from the Indian Meteorological Department (IMD), and the relative humidity from the NCAR/NCEP reanalysis (Fig. S19 in Supplemental Information). We found that the heat index results remain consistent across these two types of datasets, where the first dataset consists of variables from the reanalysis alone and the second dataset consists of a combination of variables from IMD and the reanalysis. However, the latter dataset issues ‘Extreme Danger’ warning during dry spells (i.e., HI (Before)), resulting from the underestimation of long-term mean annual temperature for our study region by reanalysis products^25,26^. Overall, our analysis successfully concludes that during the pre- and the post-monsoon seasons, rain is the most-efficient, if not the only, driver in providing physical relief from scorching heat.

## Discussion

The current study provides a spatio-temporal analysis of the effect of rainfall in decreasing the high maximum and minimum temperatures across India resulting from the persistence of long dry spells. A rainfall event is able to reduce the maximum temperature by 5 °C, primarily across the northern and the northeastern parts of the country, which experience frequent thunderstorms and cyclonic activities beyond the monsoon seasons. Our study clearly indicates that heavy rain is more effective during the pre-monsoon than a low rain, irrespective of the length of the dry spell. In addition, a statistically significant long-term trend in the magnitude of temperature drop is uncommon. Across the Ganga basin, the amount of precipitation and the change in the temperature are negatively correlated, while the impact of rain on *Tmax* is stronger than its impact on the *Tmin*. However, the statistically significant correlations across grid cells are not globally significant. Finally, we conclude that although, rain causes a decrease in the temperature, the physical relief from the rainfall is not substantial during the pre-monsoon season; however, rain does bring a bio-meteorological relief during the post-monsoon season by lowering the heat index warning from ‘caution’ to ‘normal’. So, interestingly, the empirical belief that rain provides respite from heat holds true only for post-monsoon months.

The current study defines multiple scenarios (S1 to S9) such that the definition of scenarios can be extended to analyze rainfall-temperature dynamics of any geographical region. It could issue meaningful insights on the role of rainfall in providing bio-meteorological relief particularly for tropical and sub-tropical countries where high temperatures and rainfall events are common weather conditions. In particular, similar approach is adequate for regions such as Amazon, South Atlantic, Southwest China Eastern United States, West Africa, South Australia, and Southeast Asia since these regions experience distinctive dry and wet spell characteristics^27–29^. However, caution must be exercised in individual characterization of dry spell duration and rainfall thresholds. Spatial distribution of climatological features related to dry spell durations and rainfall events must be taken in to account while defining the scenarios; which might even lead to an inclusion (elimination) of an additional (existing) scenario depending on the geographic location. Note that, the current study considers seasonality in rainfall amount; however, it does not consider the seasonality on dry spell lengths. Therefore, seasonality in rainfall and dry spell duration is partially addressed by the current study. Rainfall amounts and dry spell durations are categorized following three former studies^13–15^. These studies indicate that the seasonality in dry spell duration is not significant beyond monsoonal months. Rather, spatial variation of dry spell lengths are substantial^15^. The current study is designed such that season-to-season variations in dry spell affects the variability of the change in temperature, not the expectation. Temperature anomalies (departures from the average *Tmin* or *Tmax* for that calendar date) could have been estimated in place of the change in the temperature to understand the shower effect. However, anomalies would not provide the instantaneous impact of rainfall on current temperature. Also, anomalies will reflect the stochasticity of the process (natural variability) instead of providing an explanation of the change in the temperature. Therefore, the current study indicates the need of multi-site continuous monitoring of precipitation, temperature and relative humidity to understand shower effect with a better clarity.

A major future application of the current study is envisioned in the incorporation of the current findings in a daily weather forecast system to achieve improved accuracies in predicting surface air temperature. The idea is to model the change in the temperature (or change in the heat index) based on a rainfall forecast conditioned on the length of the dry spell the rain succeeds. This can be achieved by either developing a categorical regression model^30^ or by integrating current findings with a stochastic weather generator^31^. This future analysis is expected to influence the monsoonal crop-production and fruit farming by helping farmers to take necessary irrigation and harvesting decisions based on the rainfall amount and the change in the temperature. Additionally, a model to forecast heat index will help improve the interpretability of the daily weather forecast. Consequently, it will improve preparedness for extreme heat/temperature situations, leading to fewer incidents of heat stroke and dehydration-related illnesses^32^. Two major criticisms of the current study arise from the facts that (i) The heat index was proposed by the National Weather Service which may not be applicable for Indian context and, (ii) The study ignores other local-scale drivers such as aerosol, wind speed and wind direction that might have a direct impact on the change in the temperature. In recent years, IMD has started issuing heat wave warnings for April, May and June based on two indices-heat index and the comfort index^33^. The two indices are updated three-hourly by combining air temperature and relative humidity; although the actual calculation of the two indices are not made public by the IMD. The department issues three heat wave warnings (yellow, orange and red to represent be updated, be prepared and take action, respectively) with distinctive threshold values for the plains, the coastal and the hilly regions^34^. Once released, a future study related to the shower effect must consider the indices developed by the IMD. However, it is not uncommon to use bio-meteorological indices developed in one country for a different region. Dash *et al*.^35^, estimated the heat index (NSW) along with three other heat indices for four Indian cities using the observed and climate model data, and found that the four indices exhibit similar inter-annual variability and monthly mean values. Second major criticism of the current study is related to drivers other than rainfall that cause a change in the temperature. One of the novelties of the definition of the nine scenarios is that rainfall automatically emerges as the logical and strongest predictor for changes in temperature, thus obviating the requirement of additional drivers. Consideration of aerosol, wind speed and wind direction should not alter the magnitudes of the change in the temperature. However, inclusion of other drivers can reduce the strong dependence between rainfall and the change in the temperature when a partial correlation is calculated. In conclusion, the current study provides a novel insight on the relationship between precipitation and temperature that can be further analyzed, modelled and tested under various climate scenarios. Additionally, the proposed variable (‘change in temperature’) could be incorporated in daily weather forecasting to obtain an improved accuracy. We found that although rainfall leads to a substantial decrease in the air temperature, it does not provide substantial relief from heat waves.

## Data and Methods

Gridded observed daily precipitation (in mm) values and daily maximum and minimum temperatures (in °C) at (1° × 1°) resolution across India are obtained from the IMD. For temporal consistency, the rainfall and temperature data for the period of 1951–2004 are considered for this analysis. Daily records of meteorological variables are extracted separately for the pre-monsoon and for the post-monsoon seasons. ${X}_{i,j}^{s,T}$ and ${Y}_{i,j}^{s,T}$ represents the temperature (either *Tmax* or *Tmin*) and the rainfall variables respectively at grid cell *j* (*j* = 1 … 355) on the *i*^th^ day (*i* = 1 … 92) of the season *s* (*s* = 1 for the pre-monsoon, and *s* = 2 for the post-monsoon) in the year *T* (*T* = 1… 54). If ${Y}_{i,j}^{s,T} > 2.5$ then *i*^th^ day is labelled as a wet day, otherwise *i*^th^ day is labelled as a dry day. Length of a dry spell is denoted by ${d}_{j,k}^{s,T}$, which can be estimated using the pseudo code given below (*k* is the index of the dry spell within a year).

1. Consider initial length of dry spell: ${d}_{j,k}^{s,T}=0$.
2. If *i*^th^ day is a dry day, then check if (*i* + 1) is also a dry day. If ‘yes’ then ${d}_{j,k}^{s,T}={d}_{j,k}^{s,T}+1$, else Break.
3. Change *i* $\to \,$*i* + 1, and repeat step 2.

The pseudo code may run into interruptions if (i) All days within a season are dry (ii) First day of a season is the only wet day (iii) The last day of a season is a dry day which is part of a dry spell that extends to the next season (monsoon or winter), and (iv) Last day of a season is the only wet day. The current study ignores first three situations; however, we continue with the final case since related calculations remains unaffected from the length of the dry spell. Based on following conditions, dry spell lengths are categorized as below:

If ${d}_{j,k}^{s,T} < 4$ then ${d}_{j,k}^{s,T}$ is a ‘short’ dry spell

If $4 < {d}_{j,k}^{s,T} < 14$ then ${d}_{j}^{s}$ is a ‘moderate dry spell

If ${d}_{j,k}^{s,T} > 14$ then ${d}_{j}^{s}$ is a ‘prolonged’ dry spell

If ${X}_{t,j}^{s,T}$ and ${X}_{t+1,j}^{s,T}$ are the temperatures on the last day of a dry spell (*i* = *t*) and on the first day of a wet spell (*i* = *t* + 1) respectively then the change in temperature is calculated using following equation:

1 $${\rm{\Delta }}{X}_{k,j}^{s,T}={X}_{t+1,j}^{s,T}-{X}_{t,j}^{s,T}$$

Based on following conditions, the amount of rain on day (*t* + 1) is categorized:

If ${Y}_{t+1,j}^{s,T} < {Y}_{j}^{s,40{th}\,{Percentile}}$, then (*t* + 1)^th^ day experienced a ‘low’ rain

If ${Y}_{t+1,j}^{s,T} > {Y}_{j}^{{s},{90th}\,{Percentile}}$, then (*t* + 1)^th^ day experienced a ‘heavy’ rain

Otherwise, (*t* + 1)^th^ day experienced a ‘medium’ rain.

Rain percentiles are estimated by fitting a Gamma-inverse distribution to the observed rainfall values (${{\boldsymbol{Y}}}_{j}^{s}$) at grid cell *j* for 1951–2004. Finally, the decrease in the temperatures (${\rm{\Delta }}{X}_{k,j}^{s,T}$) is labelled based on nine scenarios (from Fig. 1b). Following this, four statistics are estimated: (i) The long-term average (ii) Mann-Kendall (M-K) test^36^ (iii) Sen-slope estimator^30^, and (iv) Kendall’s rank correlation coefficient^37^. The M-K test is a rank-based test which compares subsequent data pairs to check if a monotonic increasing or a monotonic decreasing temporal trend exists in a dataset with a certain confidence level (90% confidence level with a two-tailed significance test has been considered for our analysis). If the data exhibits a monotonic temporal trend, we consider the Sen slope value to estimate the magnitude of the slope. Sen Slope is the median of all pair-wise slopes between each data value with remaining data values. Statistical significance of the Sen slope is estimated for 95% confidence level. Both of these statistics are widely applied in climate and water resources to detect temporal trends^38^. The long-term average, M-K test and the Sen slope are estimated based on the ${\rm{\Delta }}{{\boldsymbol{X}}}_{\,j}^{{\boldsymbol{s}}}$, whereas, the rank correlation is calculated between ${\rm{\Delta }}{{\boldsymbol{Y}}}_{{\boldsymbol{j}}}^{{\boldsymbol{s}}}$and ${\rm{\Delta }}{{\boldsymbol{X}}}_{\,j}^{{\boldsymbol{s}}}$. A field significance test based on false discovery rate^23,24^ is carried out to examine whether local null-hypothesis related to p-values can be rejected globally. Field significance test is considered for the trend analysis and for the Kendall’s correlation. All related computations are carried out in MATLAB^39^ and R^40^.

Additionally, to calculate the heat index (HI), gridded relative humidity (*rhum*) at 1000 mb, daily surface precipitation (*prate*) and daily maximum temperature at 2 m (*tmax*) across the Ganga basin for the period Jan/1951-Dec/2004 is obtained from NCEP/NCAR reanalysis 1 products (CDAS-1)^41^. Gridded relative humidity, precipitation and maximum temperature values are extracted for two seasons. For the current analysis, changes in *Tmax* (from Eq. 1) under nine scenarios are calculated using reanalysis products. To estimate the heat index, the current study first calculates long-term temporal averages of *rhum*, *Tmax* (on the last day of a dry spell) and the decrease in temperature on each grid cell, then, spatially averages three variables to obtain a single value for each variable over the Ganga basin. HI (Before) is calculated using average values of *rhum* and *Tmax*, while, to calculate the HI (After), the temperature in Eq. (2) is calculated by subtracting the decrease in temperature from *Tmax*. The calculation of two HI values assumes that the relative humidity remains the same on the last day of a dry spell and the first day of a wet spell. In addition, HI (Before) and HI (After) values are also calculated with relative humidity values from the reanalysis, and daily precipitation and daily maximum temperature values from the Indian Meteorological Department. National Weather Service (NWS) uses HI to issue heat warnings. It is a measure of the level of discomfort a person might feel if the outdoor conditions are imported indoors- where the person is, and the indoor temperature is varied accordingly. The simple regression equation was first proposed by Rothfusz^42^, which was later adjusted for different temperature and relative humidity conditions.

2 $$\begin{array}{rcl}\mathrm{HI}(\mathrm{apparent}\,{\rm{temperature}}\,{\rm{in}}\,^\circ F) & = & -\,42.379+2.04901523\times {\rm{T}}+10.14333127\times {\rm{RH}}\\  &  & -\,0.22475541\times {\rm{T}}\times {\rm{RH}}-0.00683783\times {\rm{T}}\times {\rm{T}}\\  &  & -\,0.05481717\times {\rm{RH}}\times {\rm{RH}}+0.00122874\times {\rm{T}}\times {\rm{T}}\times {\rm{RH}}\\  &  & +\,0.00085282\times {\rm{T}}\times {\rm{RH}}\times {\rm{RH}}\\  &  & -0.00000199\times {\rm{T}}\times {\rm{T}}\times {\rm{RH}}\times {\rm{RH}}\end{array}$$

where, T is temperature (in °F) and RH is relative humidity (in %).

Nevertheless, the current study estimates HI using an online calculator (https://www.wpc.ncep.noaa.gov/html/heatindex.shtml). Based on HI values, NWS issues four warnings- Extreme danger, Danger, Extreme caution and Caution with each having a possible health impact resulting from extreme weather conditions.

## Supplementary information

Supplementary Information for Shower Effect

## Data Availability

The datasets generated during and/or analyzed during the current study are available from the corresponding author on reasonable request.
