# Supplementary material for: Shower effect of a rainfall onset on the heat accumulated during a preceding dry spell
Source: Sci Rep. 2019 May 7;9:7011. doi: 10.1038/s41598-019-43437-7 (PMC6505530; doi:10.1038/s41598-019-43437-7)
Supplement: Supplementary file 1 — Supplementary Information for Shower Effect [file 41598_2019_43437_MOESM1_ESM.docx]

***Supplementary information for the article***

**Shower effect of a rainfall onset on the heat accumulated during a preceding dry spell**

Rajarshi Das Bhowmik^1,^^*^, Bihu Suchetana^2^, Mengqian Lu^3, *^

^1^Department of Civil Engineering, Indian Institute of Science, Bangalore-560012 India.

^2^Interdisciplinary Center for Water Research, Indian Institute of Science, Bangalore-560012, India

^3^Department of Civil and Environmental Engineering, The Hong Kong University of Science and Technology, Clear Water Bay, Hong Kong SAR.

***^*^Corresponding Author:*** *Rajarshi Das Bhowmik (rajarshidb@iisc.ac.in) & Mengqian Lu (mengqian.lu@ust.hk)*

------------------------------------------------------------------------------------------------------------------------

**Contents:**

**Figures** S1 to S19…………………………………………………………………………….1-20

**Tables** S1 to S4………………………………………………………………………………21-24


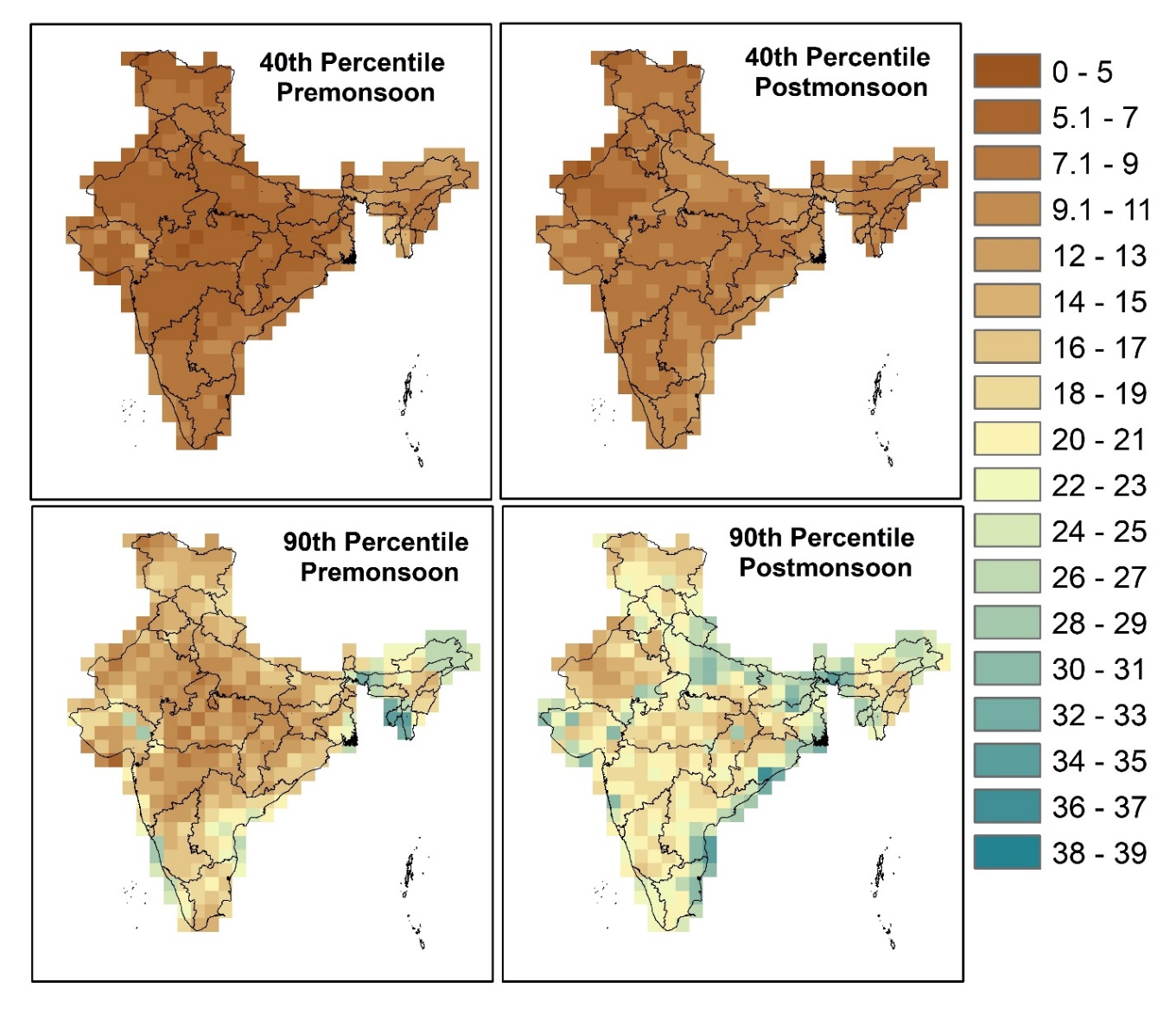


**Figure S1:** 40^th^ and 90^th^ percentile rainfall amounts (in mm/day) for grid cells across India during pre- and post-monsoon seasons.


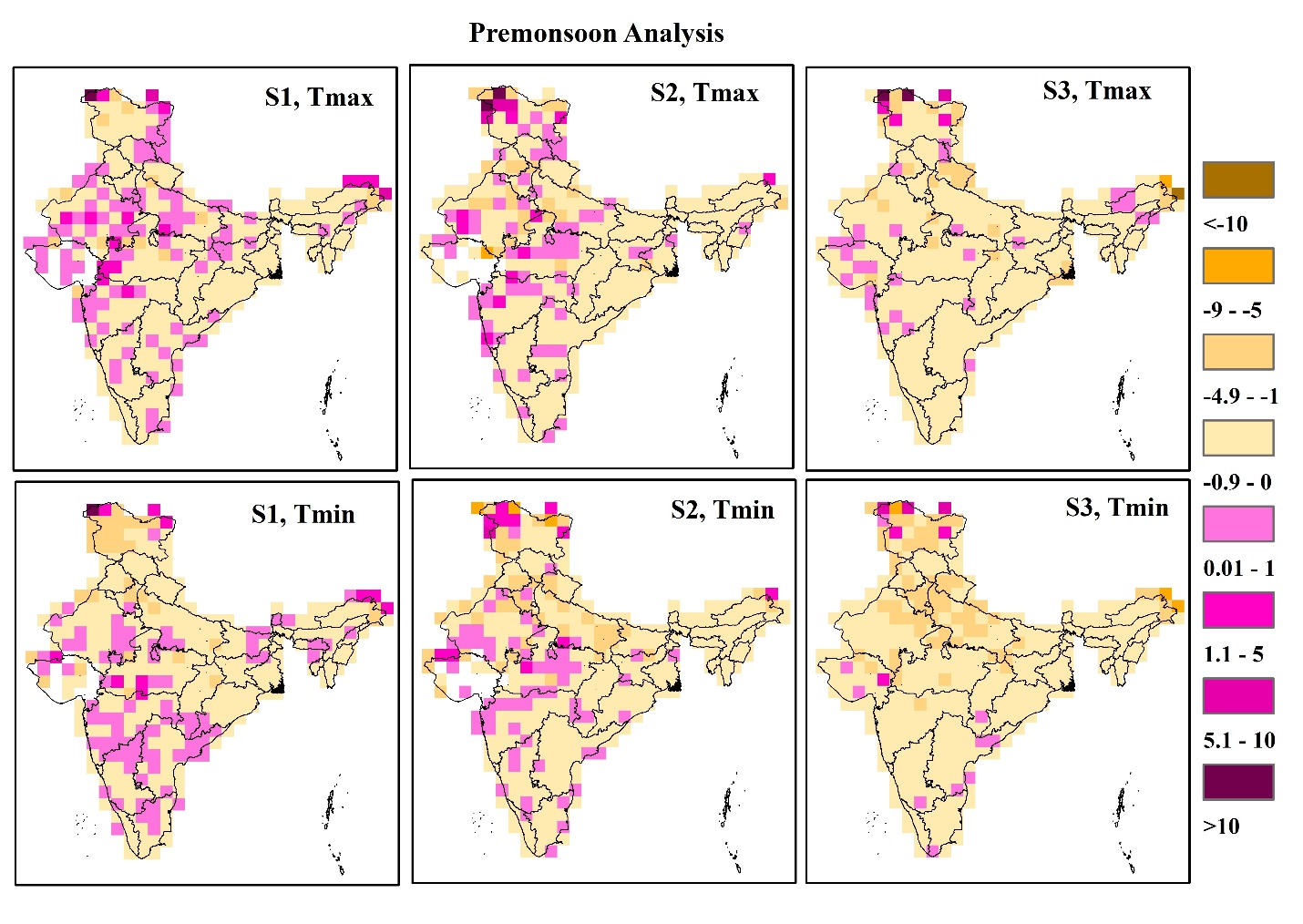


**Figure S2.** Average change in the temperature (*Tmax* and *Tmin*, in °C) across India under three different scenarios (S1, S2 and S3) for the pre-monsoon seasons, 1951-2004.


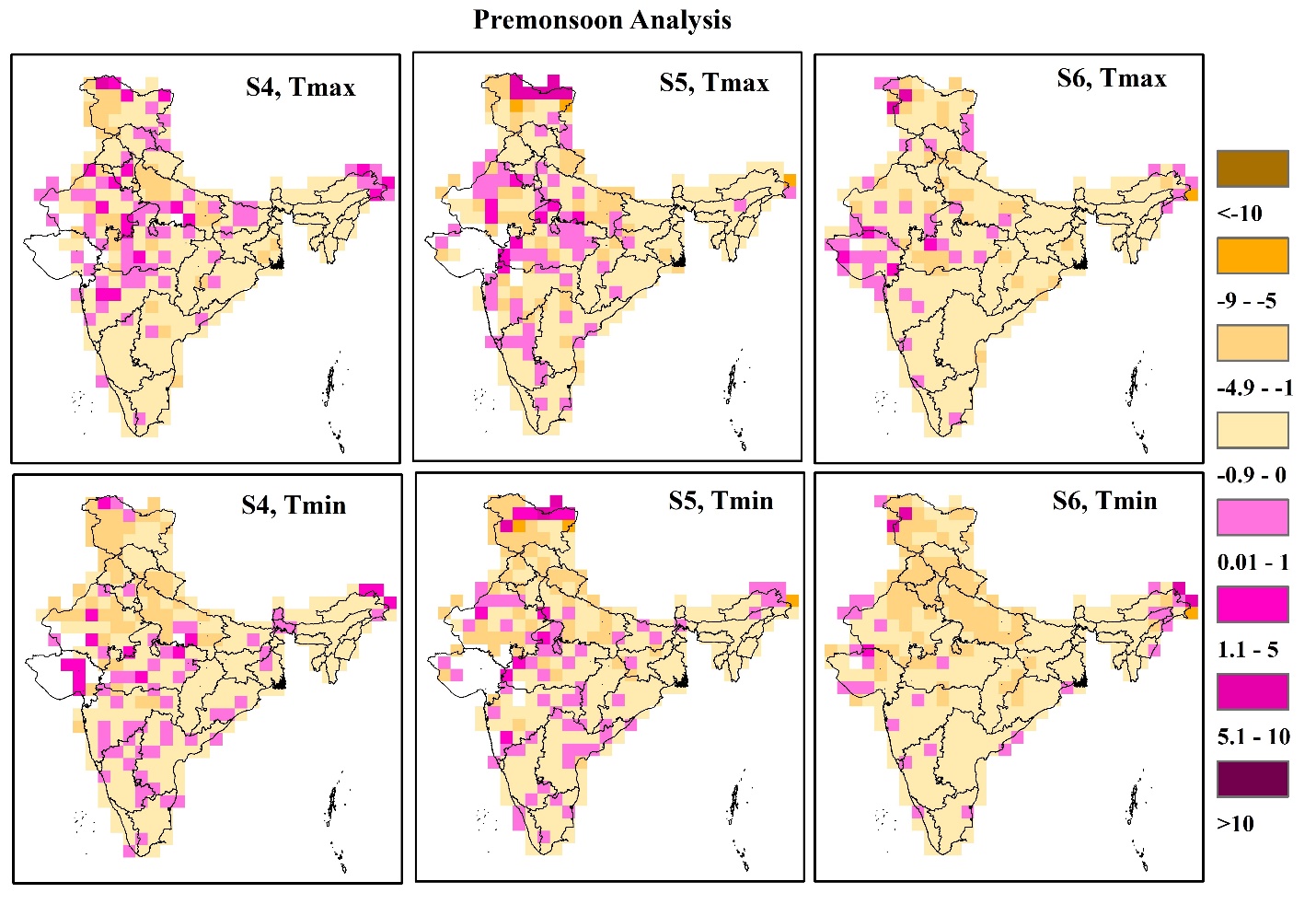


**Figure S3.** Average change in the temperature (*Tmax* and *Tmin, in °C*) across India under three different scenarios (S4, S5 and S6) for the pre-monsoon seasons, 1951-2004.


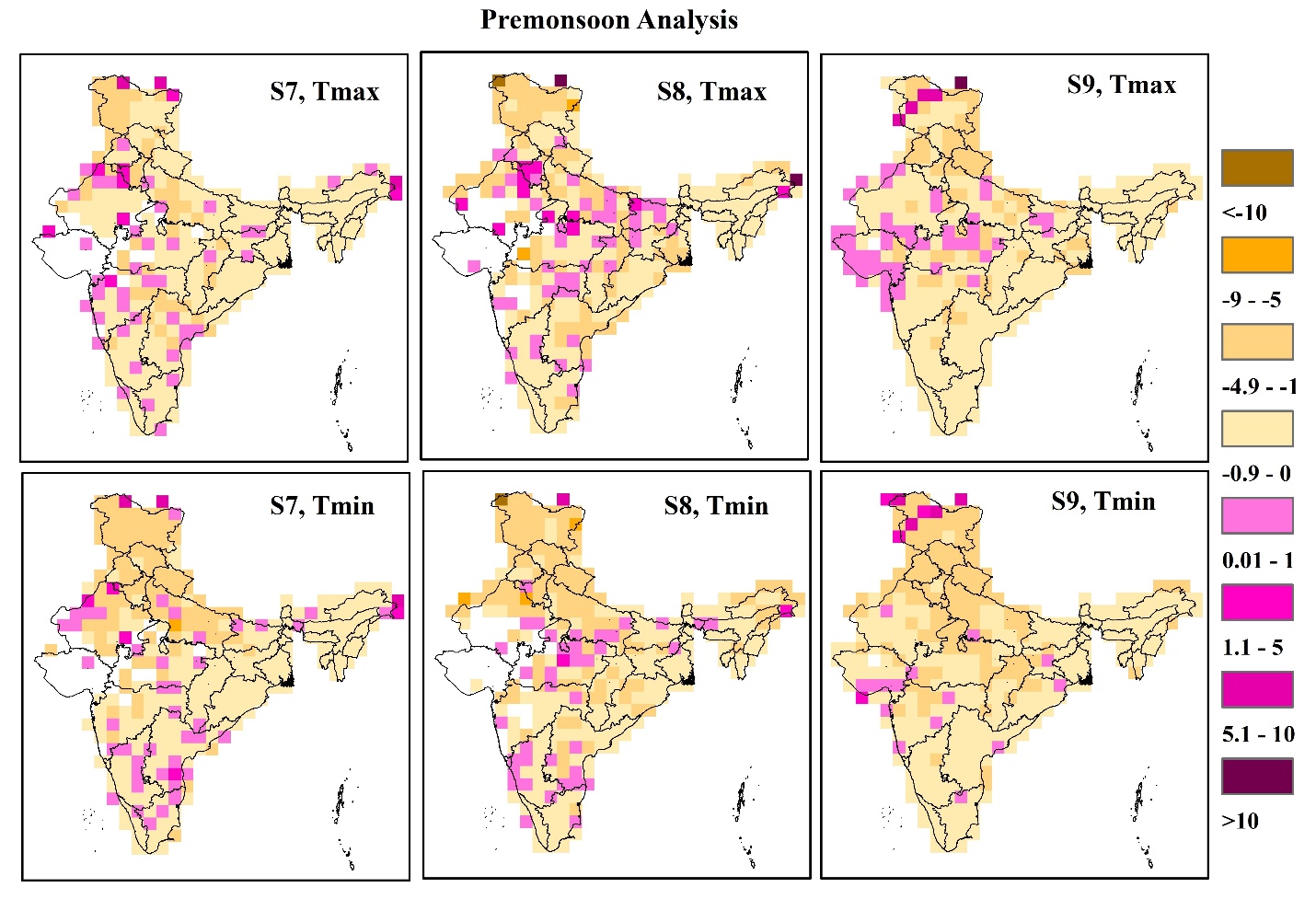


**Figure S4.** Average change in the temperature (*Tmax* and *Tmin*, in °C) across India under three different scenarios (S7, S8 and S9) for the pre-monsoon seasons, 1951-2004.


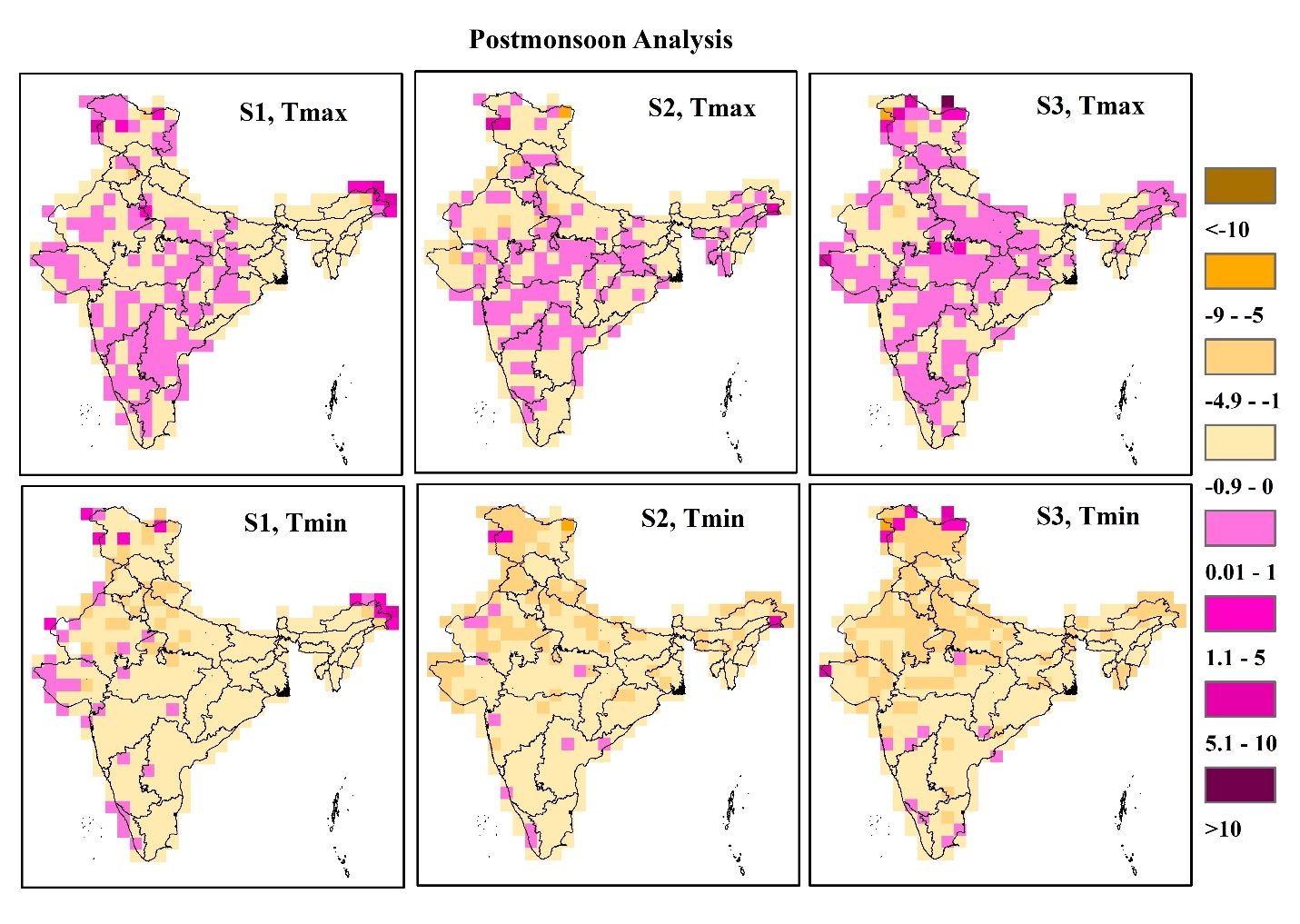


**Figure S5.** Average change in the temperature (*Tmax* and *Tmin*, in °C) across India under three different scenarios (S1, S2 and S3) for the post-monsoon seasons, 1951-2004.


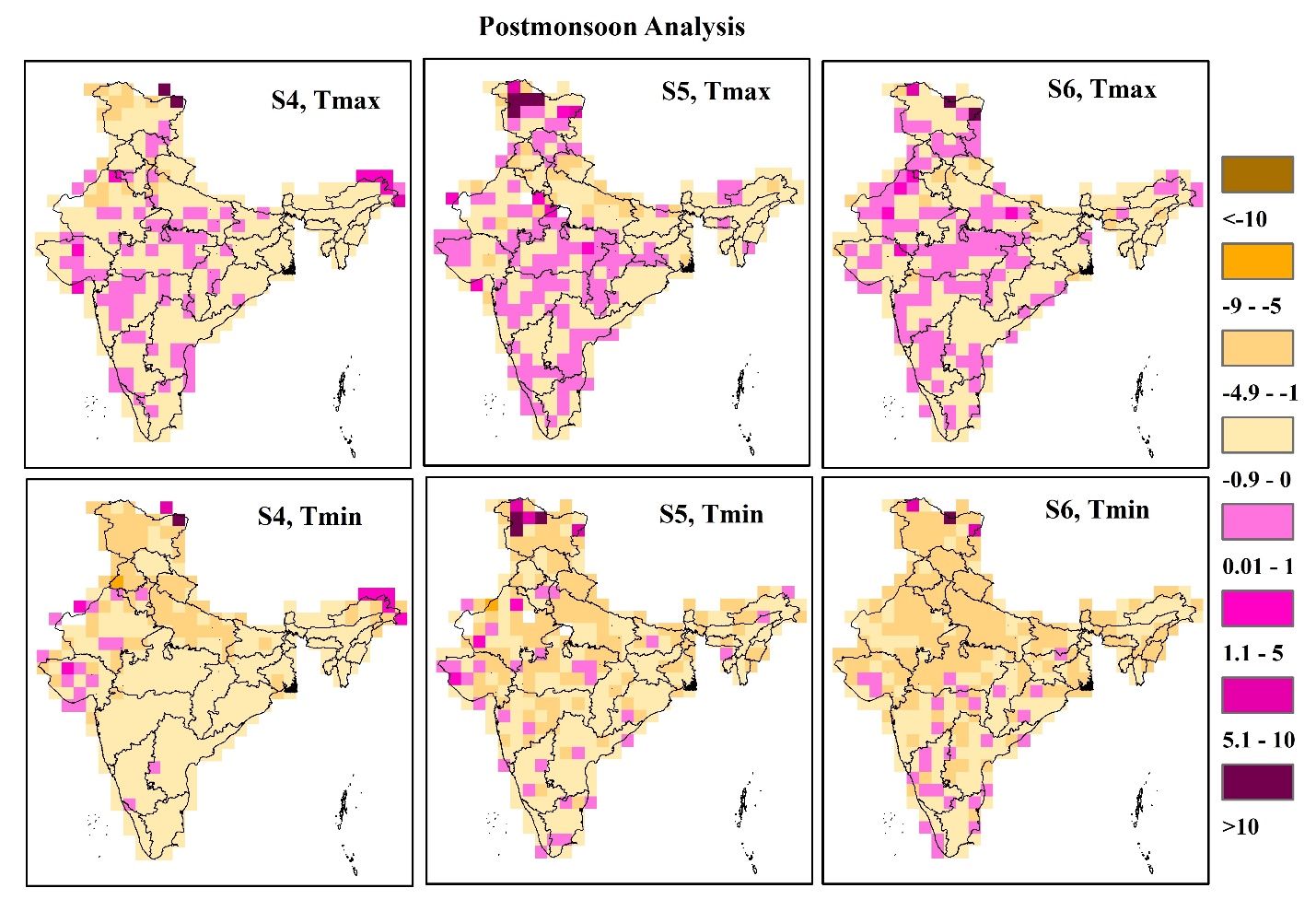


**Figure S6.** Average change in the temperature (*Tmax* and *Tmin*, in °C) across India under three different scenarios (S4, S5 and S6) for the post-monsoon seasons, 1951-2004.


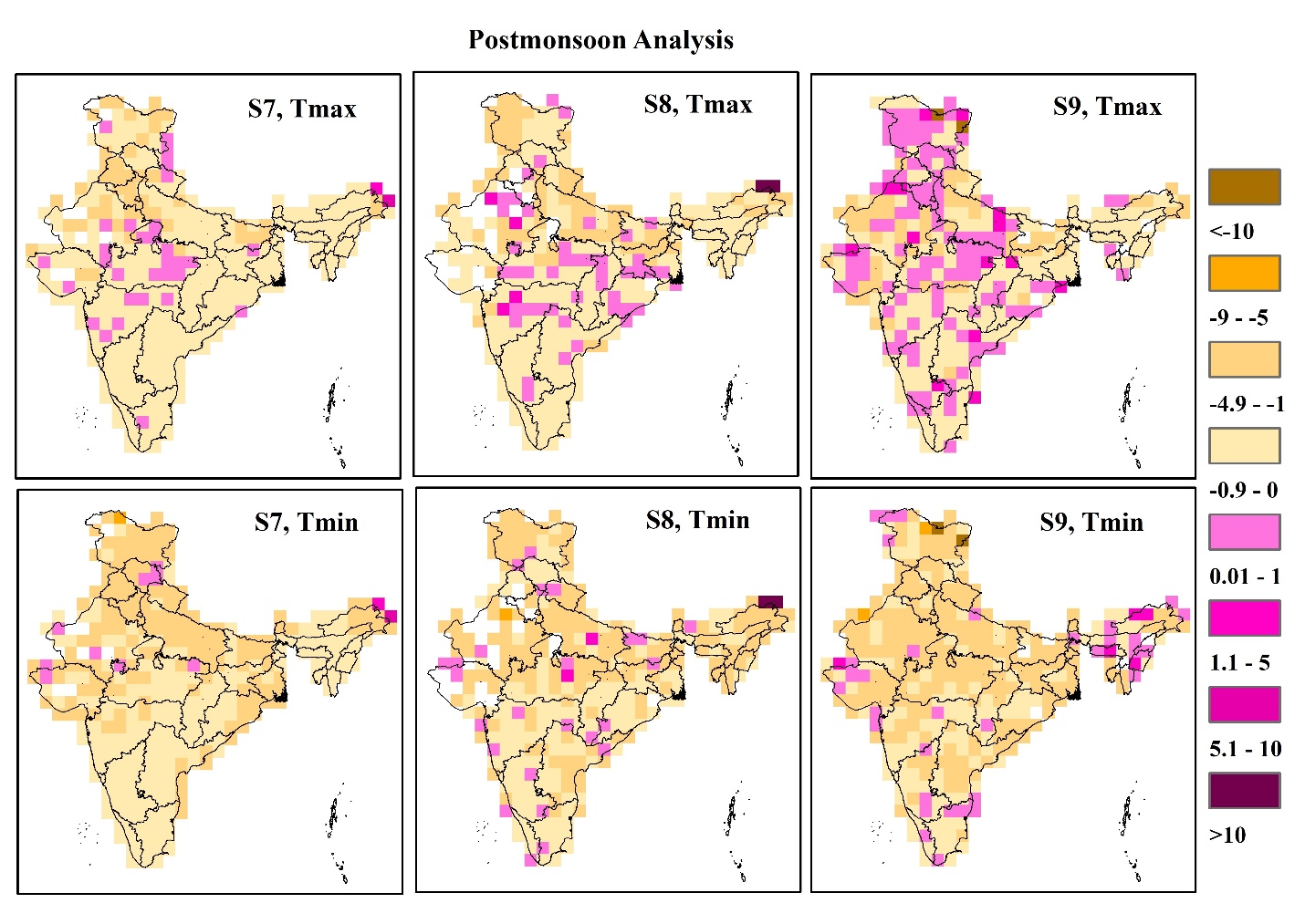


**Figure S7.** Average change in the temperature (*Tmax* and *Tmin*, in °C) across India under three different scenarios (S7, S8 and S9) for the post-monsoon seasons, 1951-2004.


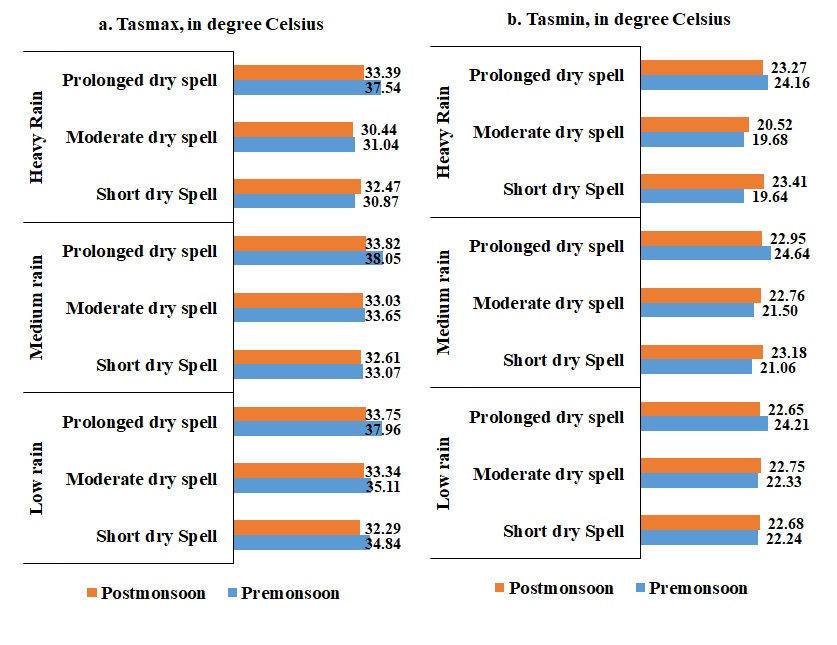


**Figure S8:** All India average of mean *Tmax* and *Tmin* (in °C) during different dry spell lengths that ended in three categories of rain events. Mean *Tmax* and the mean *Tmin* during a dry spell increases with the increase in dry spell duration. All differences in mean *Tmax* (or in mean *Tmin*) between two types of dry spells are statistically significant. A non-parametric bootstrap algorithm (with replacement) is applied to check whether two mean values under two scenarios belong to same population; i.e., statistical significance of the difference in mean values.


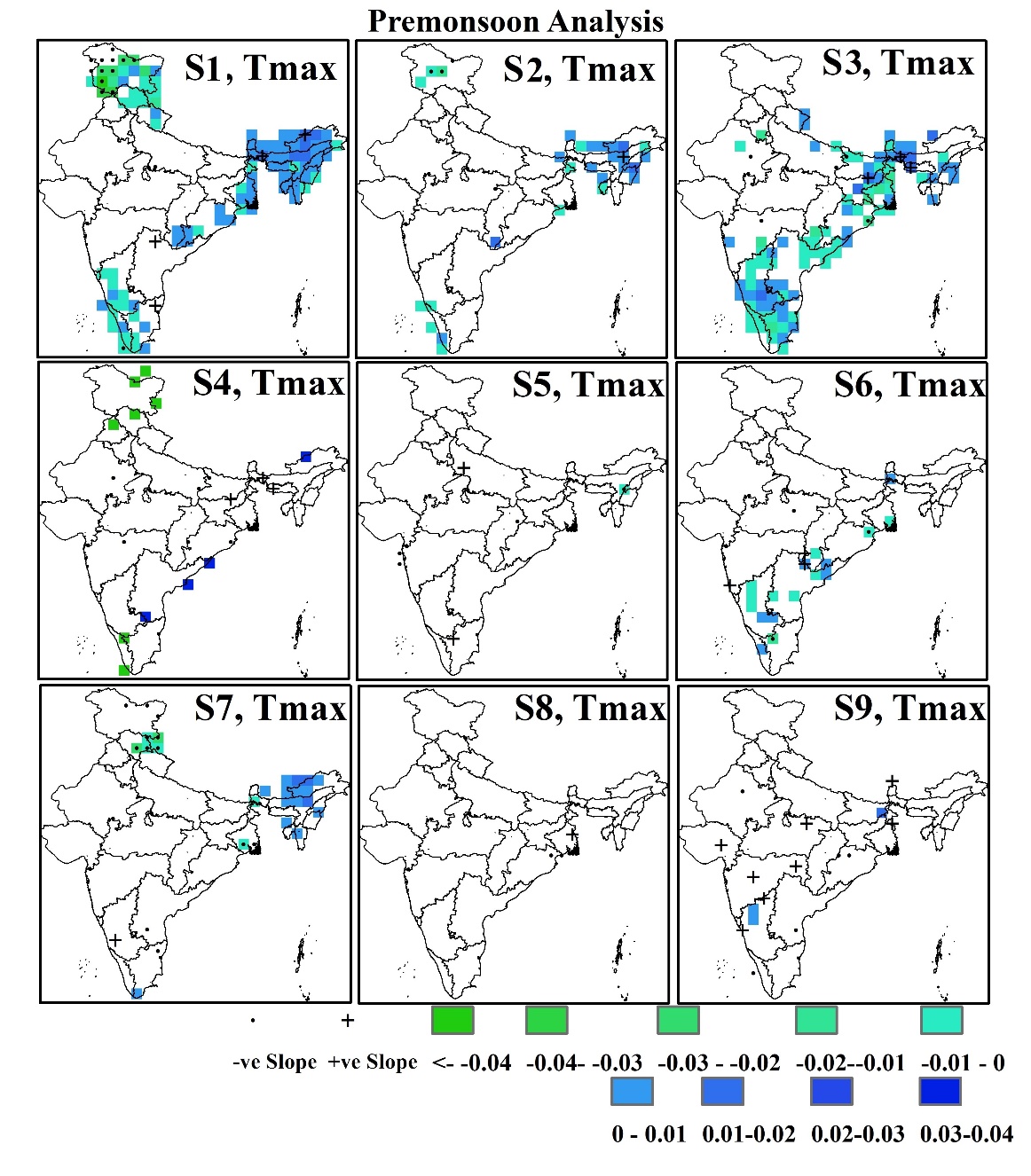


**Figure S9.** Temporal trend in the drop in the temperature (*Tmax*) for the Pre-monsoon seasons, 1951-2004. Grid points that exhibit either statistically significant positive or statistically significant negative trends, based on the Mann-Kendall test, are marked. Temporal trend is estimating following Sen slope estimator.


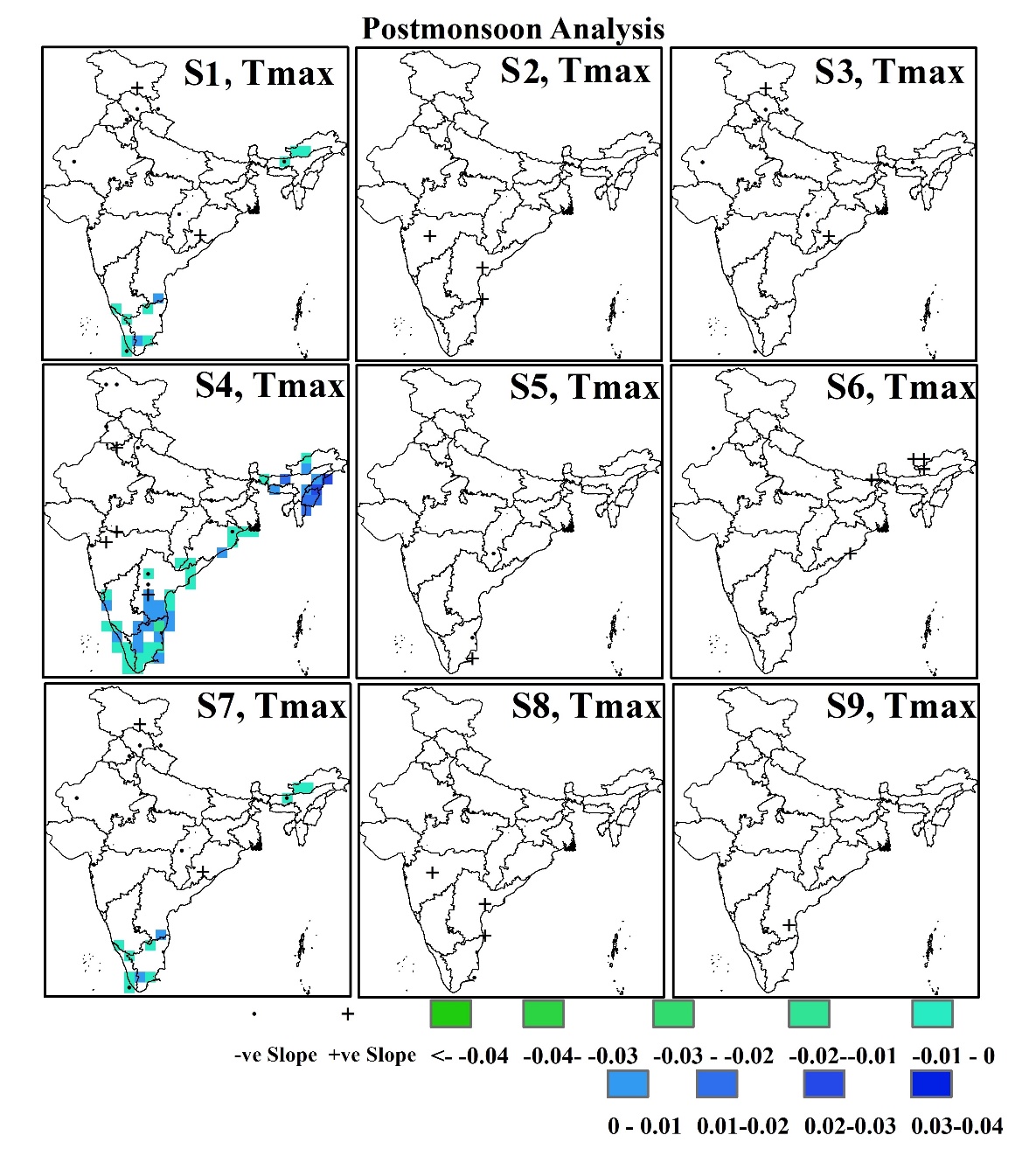


**Figure S10.** Temporal trend in the drop in the temperature (*Tmax*) for the Post-monsoon seasons, 1951-2004. Grid points that exhibit either statistically significant positive or statistically significant negative trends, based on the Mann-Kendall test, are marked. Temporal trend is estimating following Sen slope estimator.


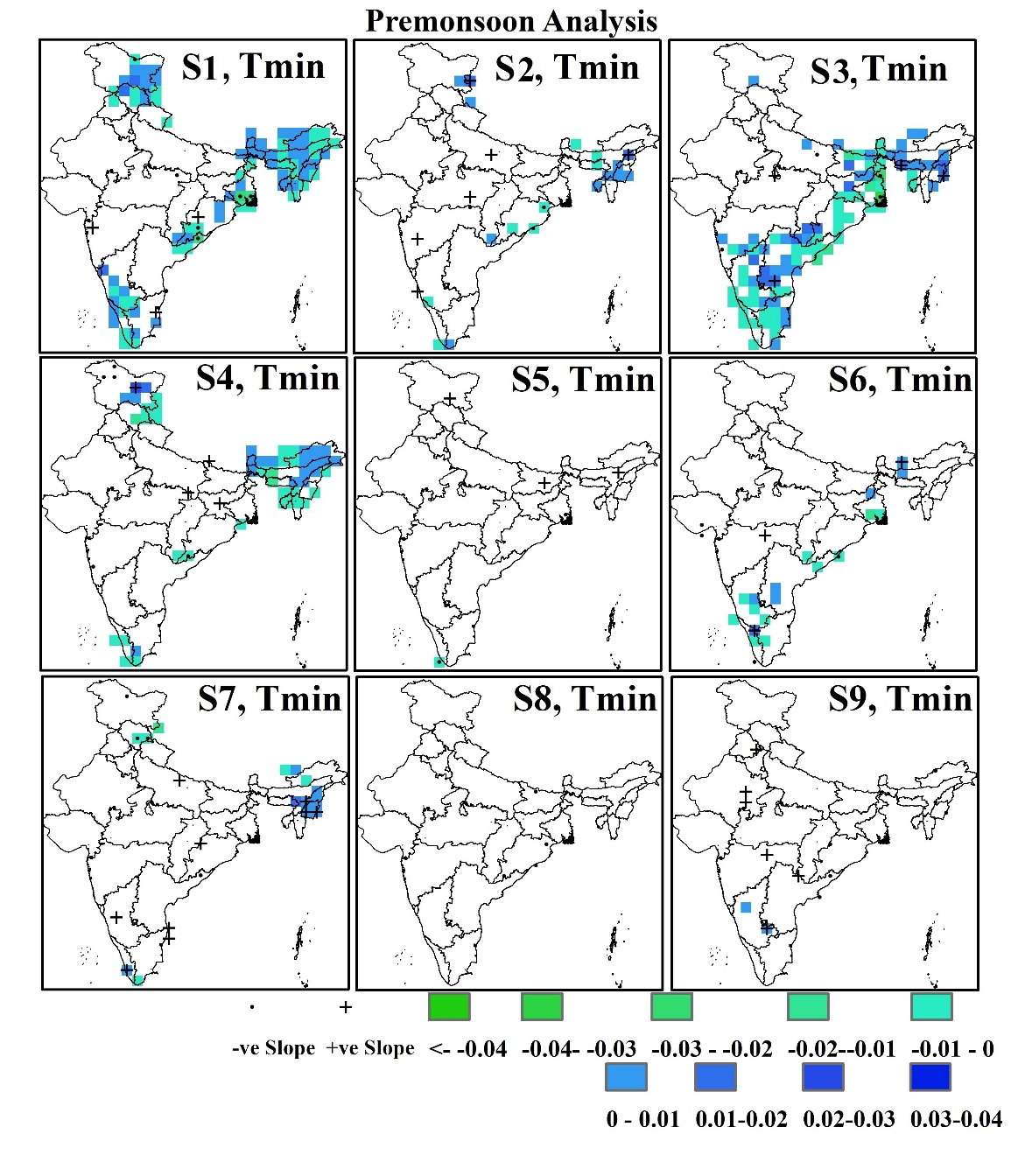


**Figure S11.** Temporal trend in the drop in the temperature (*Tmin*) for the Pre-monsoon seasons, 1951-2004. Grid points that exhibit either statistically significant positive or statistically significant negative trends, based on the Mann-Kendall test, are marked. Temporal trend is estimating following Sen slope estimator.


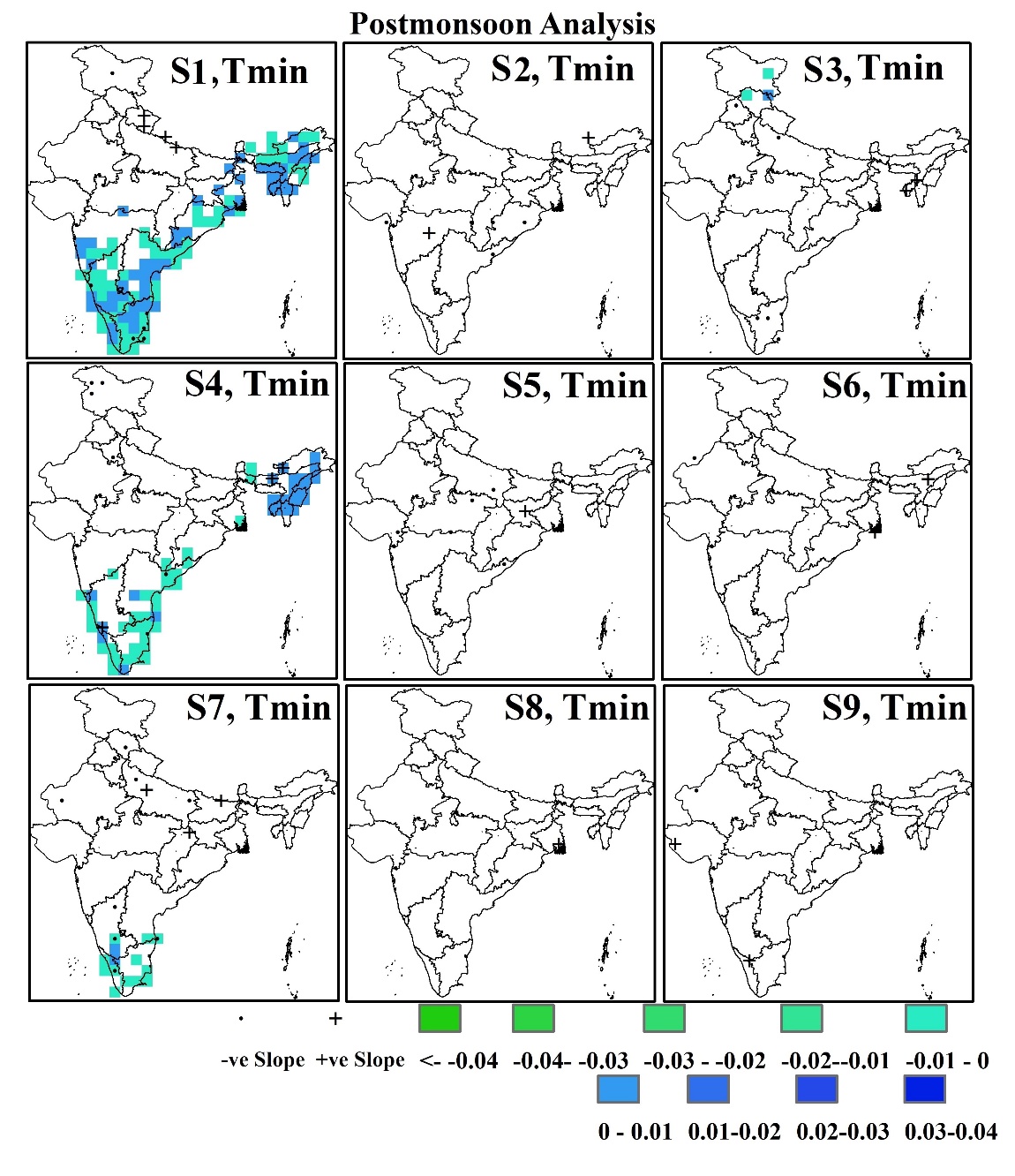


**Figure S12.** Temporal trend in the drop in the temperature (*Tmin*) for the Post-monsoon seasons, 1951-2004. Grid points that exhibit either statistically significant positive or statistically significant negative trends, based on the Mann-Kendall test, are marked. Temporal trend is estimating following Sen slope estimator.


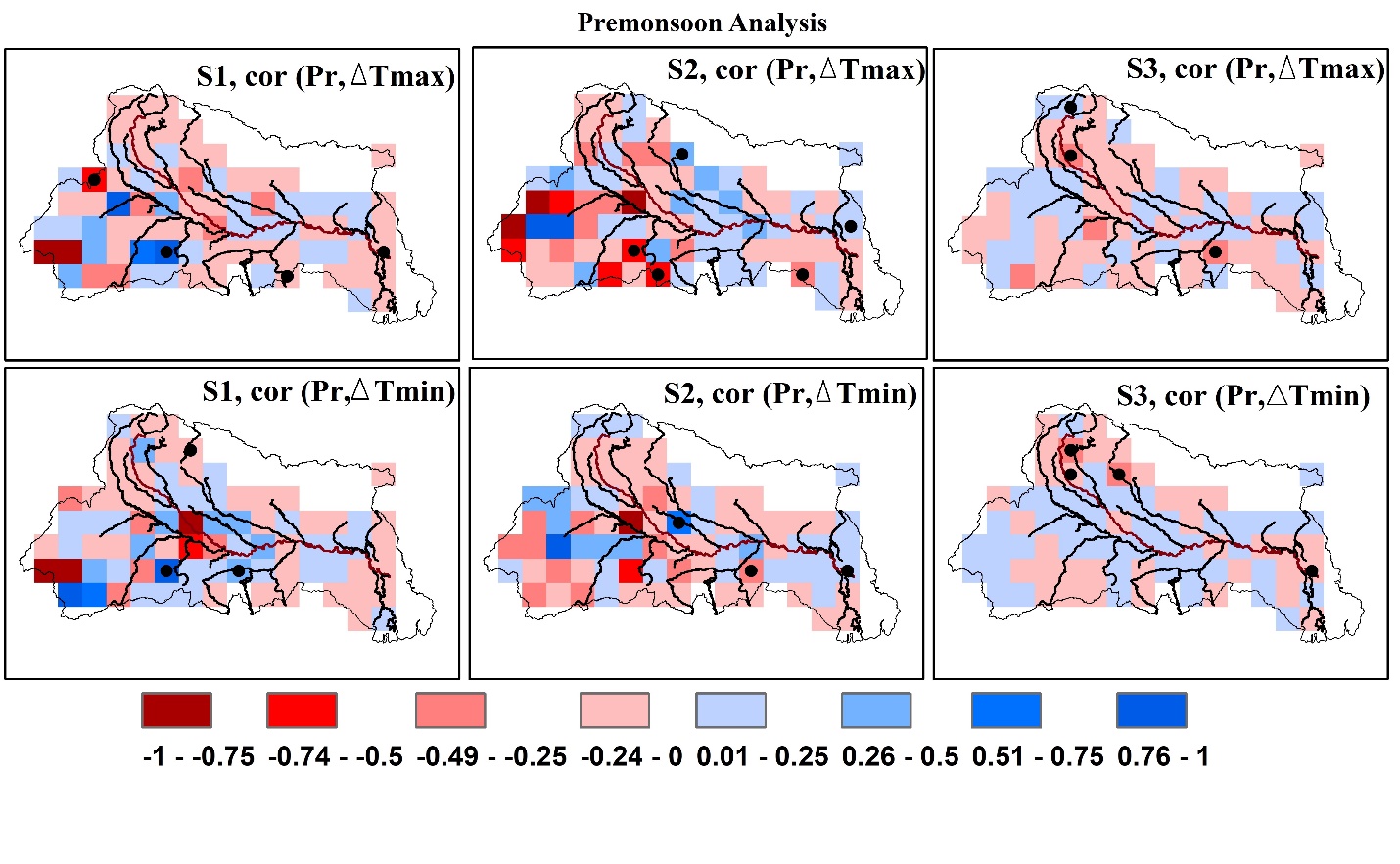


**Figure S13.** Maps show Kendall’s rank correlation coefficient between the change in the temperature (*Tmax* and *Tmin*) and the rainfall amount on the wet day succeeding a dry spell under scenarios S1, S2 and S3 for pre-monsoon seasons. Results are presented over the Ganga Basin. Statistically significant grid points are marked with black circles.


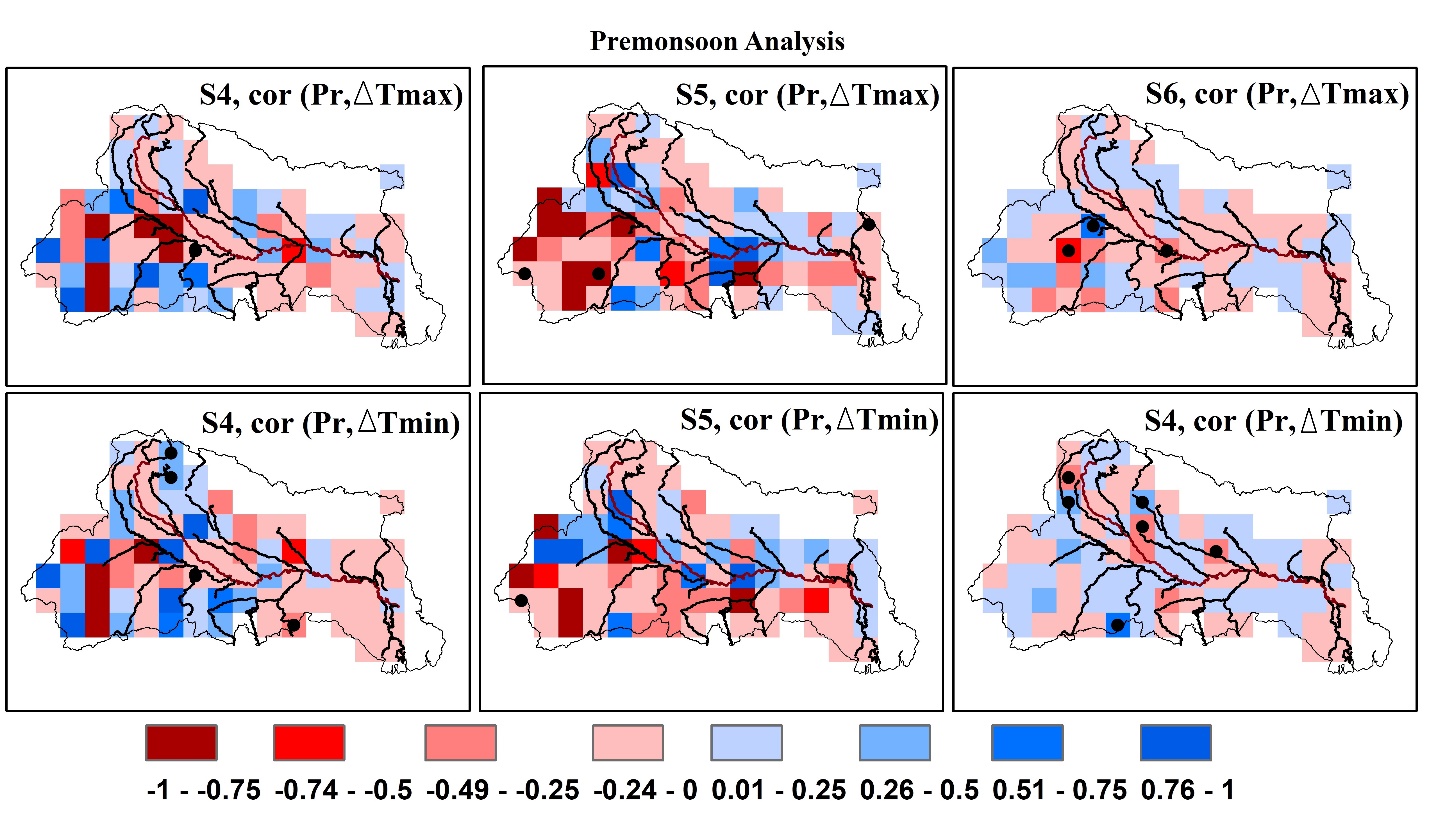


**Figure S14.** Maps show Kendall’s rank correlation coefficient between the change in the temperature (*Tmax* and *Tmin*) and the rainfall amount on the wet day succeeding a dry spell under scenarios S4, S5 and S6 for pre-monsoon seasons. Results are presented over the Ganga Basin. Statistically significant grid points are marked with black circles.


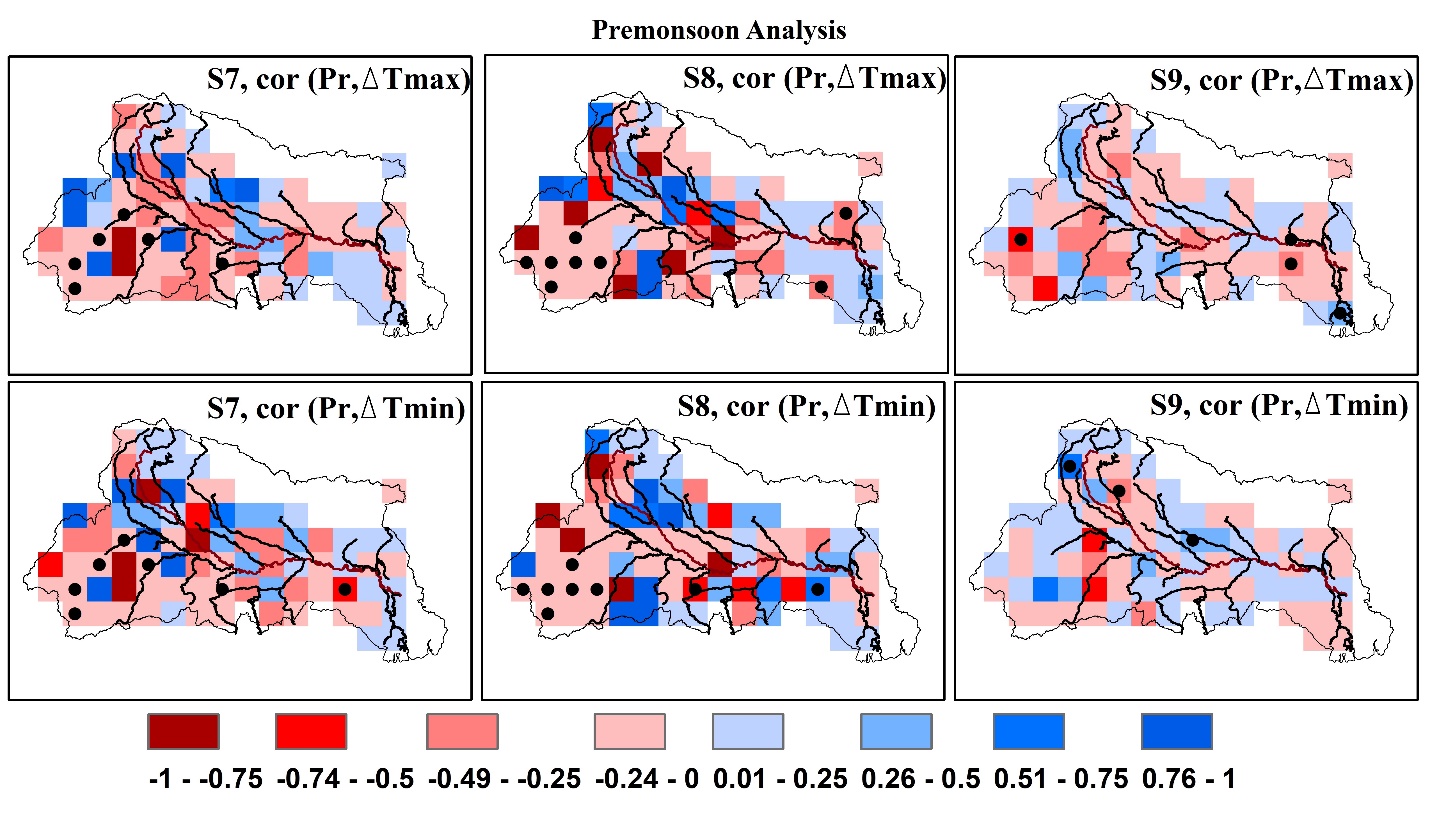


**Figure S15.** Maps show Kendall’s rank correlation coefficient between the change in the temperature (*Tmax* and *Tmin*) and the rainfall amount on the wet day succeeding a dry spell under scenarios S7, S8 and S9 for pre-monsoon seasons. Results are presented over the Ganga Basin. Statistically significant grid points are marked with black circles.


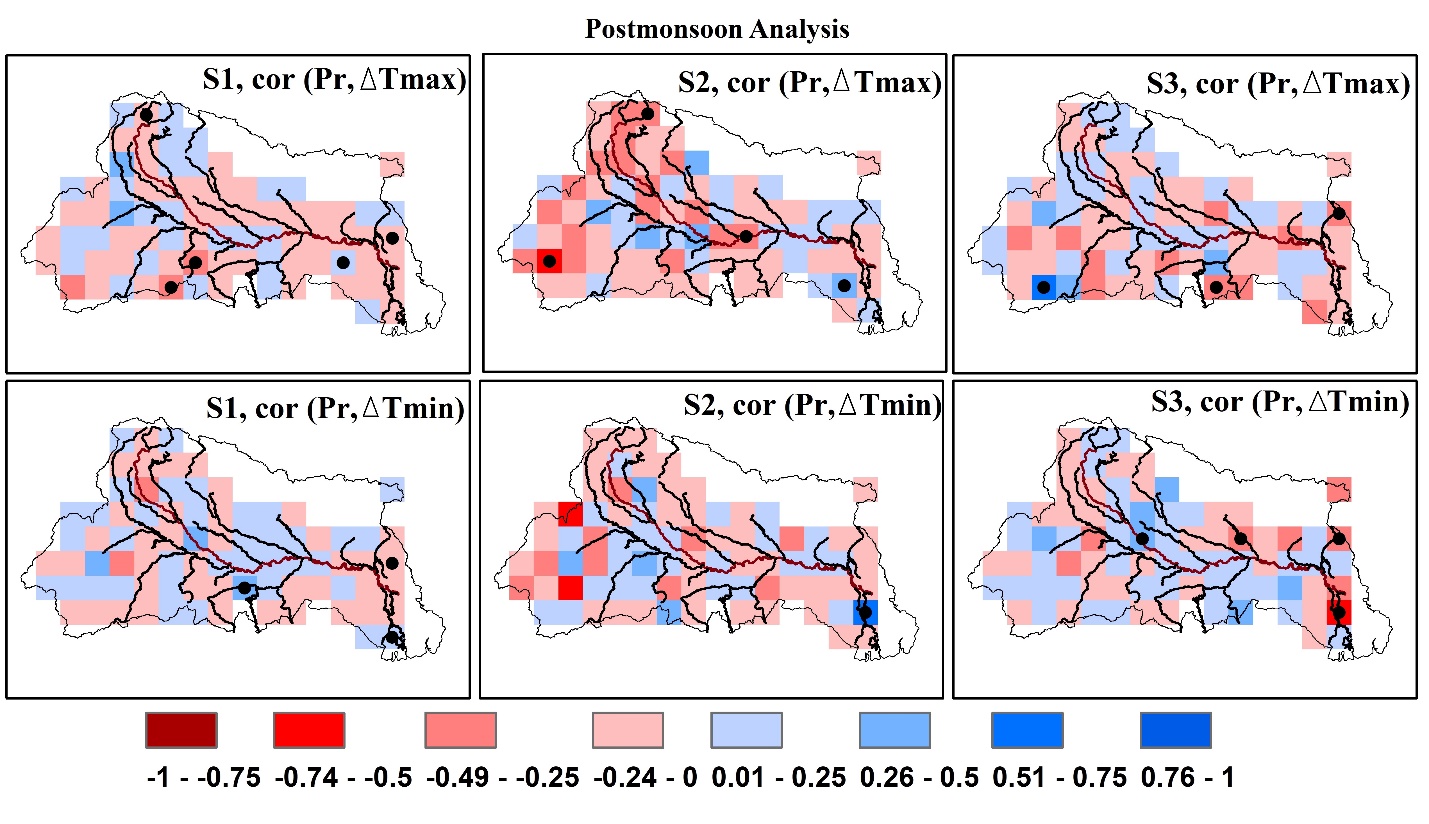


**Figure S16.** Maps show Kendall’s rank correlation coefficient between the change in the temperature (*Tmax* and *Tmin*) and the rainfall amount on the wet day succeeding a dry spell under scenarios S1, S2 and S3 for post-monsoon seasons. Results are presented over the Ganga Basin. Statistically significant grid points are marked with black circles.


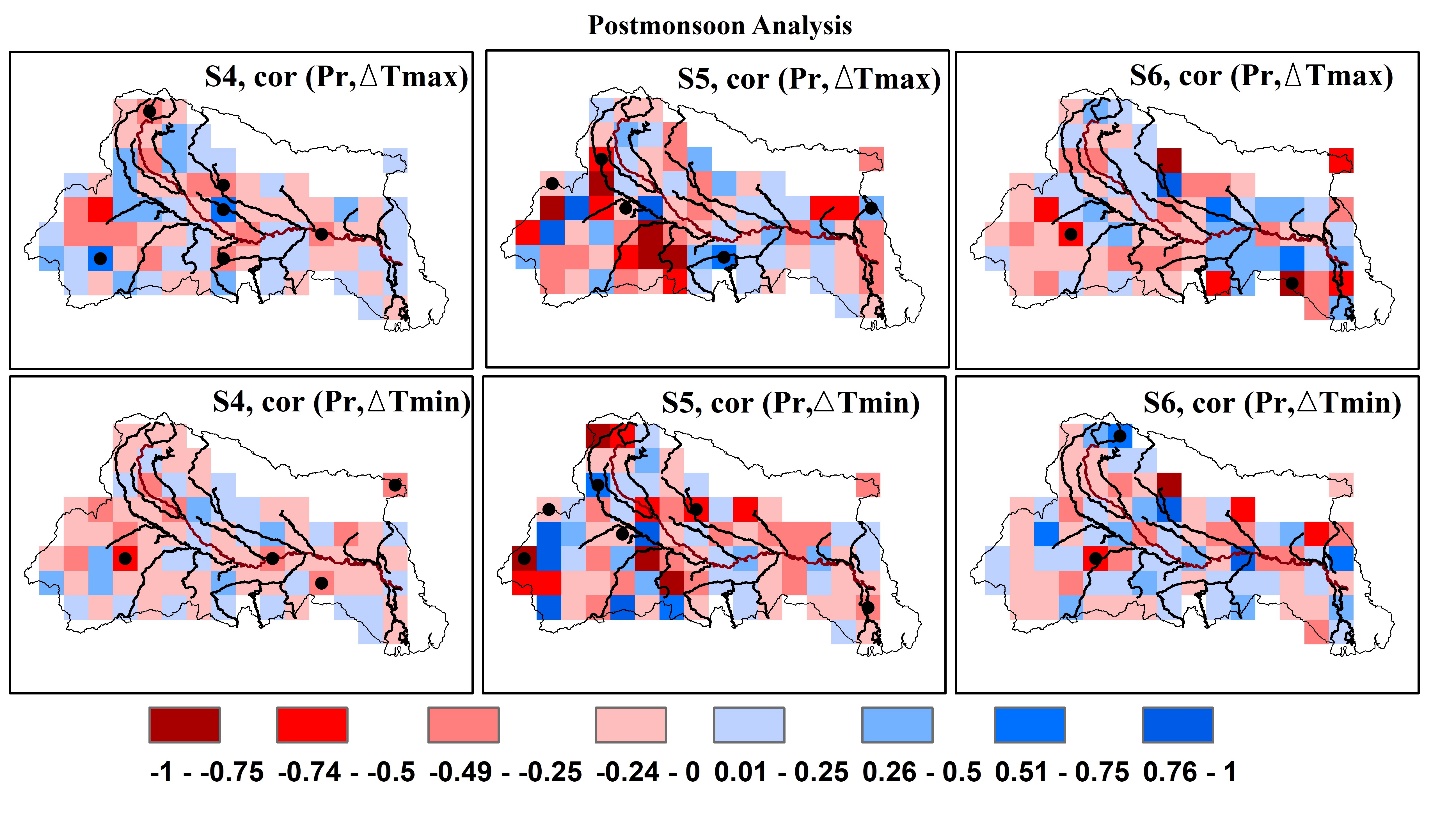


**Figure S17.** Maps show Kendall’s rank correlation coefficient between the change in the temperature (*Tmax* and *Tmin*) and the rainfall amount on the wet day succeeding a dry spell under scenarios S4, S5 and S6 for post-monsoon seasons. Results are presented over the Ganga Basin. Statistically significant grid points are marked with black circles.


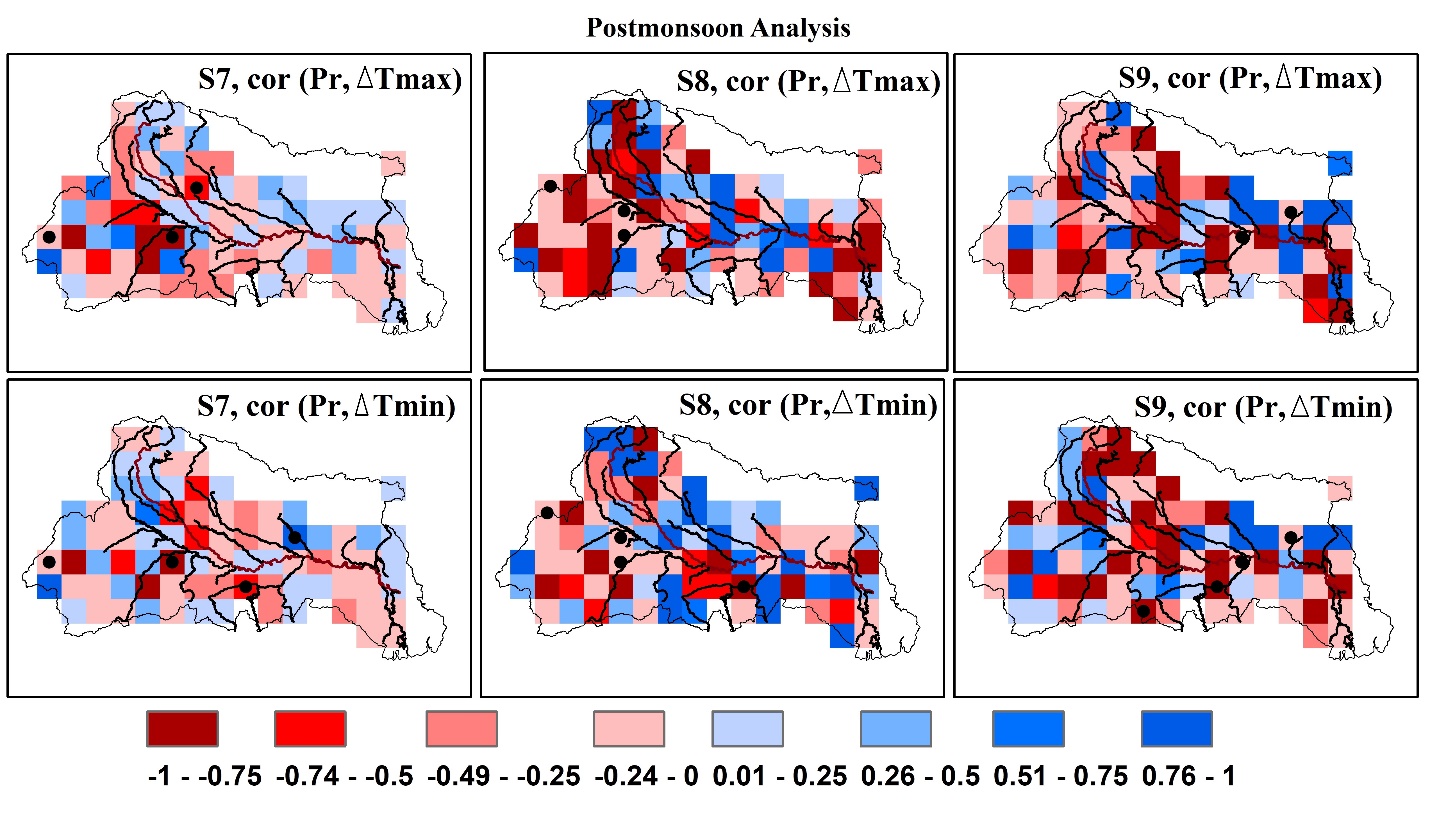


**Figure S18.** Maps show Kendall’s rank correlation coefficient between the change in the temperature (*Tmax* and *Tmin*) and the rainfall amount on the wet day succeeding a dry spell under scenarios S7, S8 and S9 for post-monsoon seasons. Results are presented over the Ganga Basin. Statistically significant grid points are marked with black circles.


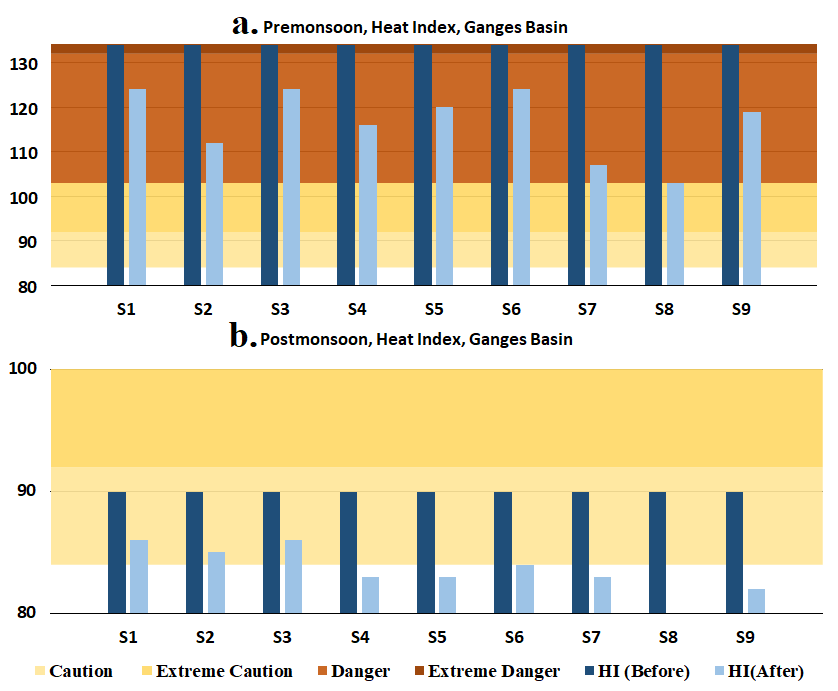


**Figure S19.** Heat Index (HI) values in ºF on the last day of the dry spell (HI-Before) and on the first day of a wet spell that succeeds the dry spell (HI-After) are presented as the bars. Background colors represents four HI warnings, ranging from caution to Extreme danger. For the current analysis, Heat Index is calculated using the spatially average decrease in the *Tmax* values, and spatially averaged relative humidity values over the Ganga basin. Gridded relative humidity valuesare obtained from the NCEP/NCAR reanalysis project (CDAS-I), whereas gridded daily precipitation and maximum temperature values are obtained from the Indian Meteorological Departmet..

**Table S1:** Results of field significance test based on false discovery rate related to Man-Kendall test. Change in *Tmax* and change in *Tmin* during pre-monsoon season on the first day of a wet spell is considered for the analysis. Field significance test result of ‘0’ (‘1’) indicates that the null hypothesis cannot (can) be rejected globally.

|  | **Pre-monsoon** | | | | | | | |  |
| --- | --- | --- | --- | --- | --- | --- | --- | --- | --- |
|  | **S1** | **S2** | **S3** | **S4** | **S5** | **S6** | **S7** | **S8** | **S9** |
| **Tmax** | 0 | 0 | 0 | 0 | 0 | 0 | 0 | 0 | 0 |
| **Tmin** | 0 | 0 | 0 | 0 | 0 | 0 | 0 | 0 | 0 |

**Table S2:** Results of field significance test based on false discovery rate related to Man-Kendall test. Change in *Tmax* and change in *Tmin* during the post-monsoon season on the first day of a wet spell is considered for the analysis. Field significance test result of ‘0’ (‘1’) indicates that the null hypothesis cannot (can) be rejected globally.

|  | **Post-monsoon** | | | | | | | |  |
| --- | --- | --- | --- | --- | --- | --- | --- | --- | --- |
|  | **S1** | **S2** | **S3** | **S4** | **S5** | **S6** | **S7** | **S8** | **S9** |
| **Tmax** | 0 | 0 | 0 | 0 | 0 | 0 | 0 | 0 | 0 |
| **Tmin** | 0 | 0 | 0 | 0 | 0 | 0 | 0 | 0 | 0 |

**Table S3:** Results of field significance test based on false discovery rate related to Kendall’s rank correlation coefficient. Change in *Tmax* on the first day of a wet spell is considered for the analysis. Field significance test result of ‘0’ (‘1’) indicates that the null hypothesis cannot (can) be rejected globally. Ganga basin is considered for the analysis. The correlation is estimated between the amount of rain and the decrease in *Tmax* on the first day of a wet spell.

|  | **S1** | **S2** | **S3** | **S4** | **S5** | **S6** | **S7** | **S8** | **S9** |
| --- | --- | --- | --- | --- | --- | --- | --- | --- | --- |
| **Pre-monsoon** | 0 | 0 | 0 | 0 | 0 | 0 | 0 | 0 | 0 |
| **Post-monsoon** | 0 | 0 | 0 | 0 | 0 | 0 | 0 | 0 | 0 |

**Table S4:** Results of field significance test based on false discovery rate related to Kendall’s rank correlation coefficient. Change in *Tmin* on the first day of a wet spell is considered for the analysis. Field significance test result of ‘0’ (‘1’) indicates that the null hypothesis cannot (can) be rejected globally. Ganga basin is considered for the analysis. The correlation is estimated between the amount of rain and the decrease in *Tmin* on the first day of a wet spell.

|  | **S1** | **S2** | **S3** | **S4** | **S5** | **S6** | **S7** | **S8** | **S9** |
| --- | --- | --- | --- | --- | --- | --- | --- | --- | --- |
| **Pre-monsoon** | 0 | 0 | 0 | 1 | 0 | 0 | 0 | 0 | 0 |
| **Post-monsoon** | 0 | 0 | 0 | 0 | 0 | 0 | 0 | 0 | 0 |
